# The Na^+^-K^+^-ATPase alpha subunit is an entry receptor for white spot syndrome virus

**DOI:** 10.1128/mbio.03787-24

**Published:** 2025-02-18

**Authors:** Junyi Zhou, Huimin Zhang, Gaochun Wu, Yinghao Zhang, Jude Juventus Aweya, Muhammad Tayyab, Jinghua Zhu, Yueling Zhang, Defu Yao

**Affiliations:** 1 Institute of Marine Sciences and Guangdong Provincial Key Laboratory of Marine Biology, Shantou University 12386 , Shantou, China; 2 Department of Food and Human Nutritional Sciences, University of Manitoba 8664 , Winnipeg, Manitoba, Canada; 3 The Canadian Centre for Agri-Food Research in Health and Medicine, St Boniface Hospital Albrechtsen Research Centre, Winnipeg, Manitoba, Canada; Columbia University, New York, New York, USA

**Keywords:** WSSV, crustaceans, virus entry, cell receptor, Na^+^-K^+^-ATPase

## Abstract

White spot syndrome virus (WSSV) is a debilitating viral pathogen that poses a significant threat to the global crustacean farming industry. It has a wide host tropism because it uses several receptors to facilitate its attachment and entry. Thus far, not all the receptors have been identified. Here, we employed a BioID-based screening method to identify the Na^+^-K^+^-ATPase alpha subunit (*Pv*ATP1A) as a potential receptor in *Penaeus vannamei*. Although during the early stages of WSSV infection*, Pv*ATP1A was induced and underwent oligomerization, clustering, and internalization, knockdown of *Pv*ATP1A inhibited viral entry and replication. *Pv*ATP1A interacted with the WSSV envelope protein VP28 through its multiple extracellular regions, whereas synthetic *Pv*ATP1A extracellular region peptides blocked WSSV entry and replication. We showed that *Pv*ATP1A did not affect WSSV attachment but facilitated internalization via caveolin-mediated endocytosis and macropinocytosis. These findings provide a robust receptor screening approach that identified *Pv*ATP1A as an entry receptor for WSSV, presenting a novel target for the development of anti-WSSV therapeutics.

**IMPORTANCE:**

Cell surface receptors are crucial for mediating virus entry into host cells. Identification and characterization of virus receptors are fundamental yet challenging aspects of virology research. In this study, a BioID-based screening method was employed to identify the Na^+^-K^+^-ATPase alpha subunit (*Pv*ATP1A) as a potential receptor for white spot syndrome virus (WSSV) in the shrimp *Penaeus vannamei*. We demonstrated that *Pv*ATP1A interacted with the WSSV envelope protein VP28 via its multiple extracellular regions, thereby promoting viral internalization through caveolin-mediated endocytosis and macropinocytosis. Importantly, compared with previously identified WSSV receptors such as β-integrin, glucose transporter 1 (Glut1), and polymeric immunoglobulin receptor (pIgR), *Pv*ATP1A demonstrated significantly enhanced viral entry, indicating that *Pv*ATP1A is a crucial entry receptor of WSSV. This study not only presents a robust approach for screening virus receptors but also identifies *Pv*ATP1A as a promising target for the development of anti-WSSV therapeutics.

## INTRODUCTION

As obligate intracellular parasites, viruses must enter permissive host cells to replicate. This process begins with the attachment of virus particles to the host cell surface, followed by binding to specific receptors, and subsequent internalization through receptor-mediated endocytosis or membrane fusion (1). Thus, cell surface receptors play a central role in viral entry as they determine host range and tissue tropism, which are key in regulating viral pathogenicity (2). For this reason, most viruses such as hepatitis C virus (HCV) (3), human immunodeficiency virus (HIV) (4), and SARS-CoV-2 (5) have evolved to utilize multiple receptors to enter host cells. Currently, two general categories of virus receptors exist, that is, attachment factors and entry receptors (6). Attachment factors primarily bind to viruses, concentrating them on the cell surface without directly promoting virus internalization (7). The interactions between attachment factors and viruses are relatively nonspecific and involve small, negatively charged molecules such as proteins, lipids, or sugars (e.g., heparan sulfate and sialic acid). On the other hand, entry receptors, mostly transmembrane proteins, bind with virus particles with high specificity and actively facilitate virus internalization by inducing conformational changes in the virus particle and triggering cellular signaling pathways that initiate endocytosis (7). Hence, identifying virus receptors and characterizing virus-receptor interactions are essential for understanding virus infection and pathogenesis, offering potential targets for antiviral strategies.

White spot syndrome virus (WSSV), the sole member of the *Whispovirus* genus within the *Nimaviridae* family, is a large, enveloped, double-stranded DNA virus with a genomic size of approximately 300 kilobase pairs (8). Due to the exceptionally broad host range of WSSV, it infects up to 98 species and is highly infectious and lethal in penaeid shrimp, causing up to 100% mortality within 3–10 days (9, 10). Many cellular receptors that facilitate WSSV infection have been identified. In shrimp, such as *Marsupenaeus japonicus*, *Penaeus vannamei*, and *Fenneropenaeus chinensis*, integrin interacts with multiple WSSV envelope proteins to mediate viral infection (11–13). In *Penaeus monodon*, membrane proteins, including Rab7, glucose transporter 1 (Glut1), chitin-binding protein (CBP), and laminin receptor (LamR), have been shown to enhance WSSV infection by binding to viral envelope proteins (14–18). Similarly, in *P. vannamei,* WSSV uses the Bip protein as an attachment factor for infection (19). In *M. japonicus*, WSSV interacts with a soluble C-type lectin (CTL) through the VP28 protein to promote its infection (20), whereas the binding of WSSV envelope protein VP24 to the polymeric immunoglobulin receptor (pIgR) facilitates viral entry via the pIgR-CaM-clathrin-mediated endocytic pathway (21). These studies have greatly enhanced our understanding of the WSSV entry mechanism. Although the WSSV receptors Rab7, CBP, LamR, CTL, and Bip are regarded as viral attachment factors, probably because they lack transmembrane domains, β-integrin, Glut1, and pIgR are reported as transmembrane proteins that act as WSSV entry receptors (21). Interestingly , it has been shown that knockdown of β-integrin or pIgR (21), and neutralization of Glut1 using synthetic peptides (15, 18), did not significantly affect the survival of WSSV-infected shrimp. Other unidentified WSSV entry factors could account for this observation, which requires further characterization.

Although virus overlay protein binding assay (VOPBA) (14), far-Western blot (12, 13), yeast two-hybrid (16, 18), and phage display (11) are some of the commonly used screening methods for virus receptors, these techniques have limitations, including low affinity and rapid dynamic changes between virus and receptor interaction, and low solubility of transmembrane receptors. Recent developments in interaction proteomics, such as the BioID (proximity-dependent biotin identification) technique, can analyze protein-protein interactions in living cells (22–24). The BioID method, which involves fusing a promiscuous biotin ligase with proximity labeling capabilities to a bait protein, allows the enzyme to catalyze proximity-dependent biotinylation, labeling proteins that are physically close to the bait protein (25). By this, the interacting proteins can be captured using the biotin-streptavidin affinity and identified by mass spectrometry. Thus, to help us identify other key WSSV entry factors in penaeid shrimp, the current study leveraged the advantages of the BioID technique, using the abundant WSSV envelope protein VP28 as the bait protein to identify specific host surface receptors, including transmembrane proteins. Using this approach, followed by further characterization using many molecular techniques, including RNAi-mediated knockdown, immunofluorescence, flow cytometry, molecular docking, and peptide blocking, we identified the transmembrane protein, Na^+^-K^+^-ATPase alpha subunit in *P. vannamei* (*Pv*ATP1A), as a key host entry factor that interacts with WSSV VP28 protein to aid viral internalization during infection. Our results suggest a crucial role of *Pv*ATP1A as an entry receptor for WSSV infection and that it could be leveraged as a potential antiviral therapeutic target.

## RESULTS

### Nine transmembrane proteins that potentially interact with WSSV VP28 protein were identified using BioID screening

**Fig 1:**
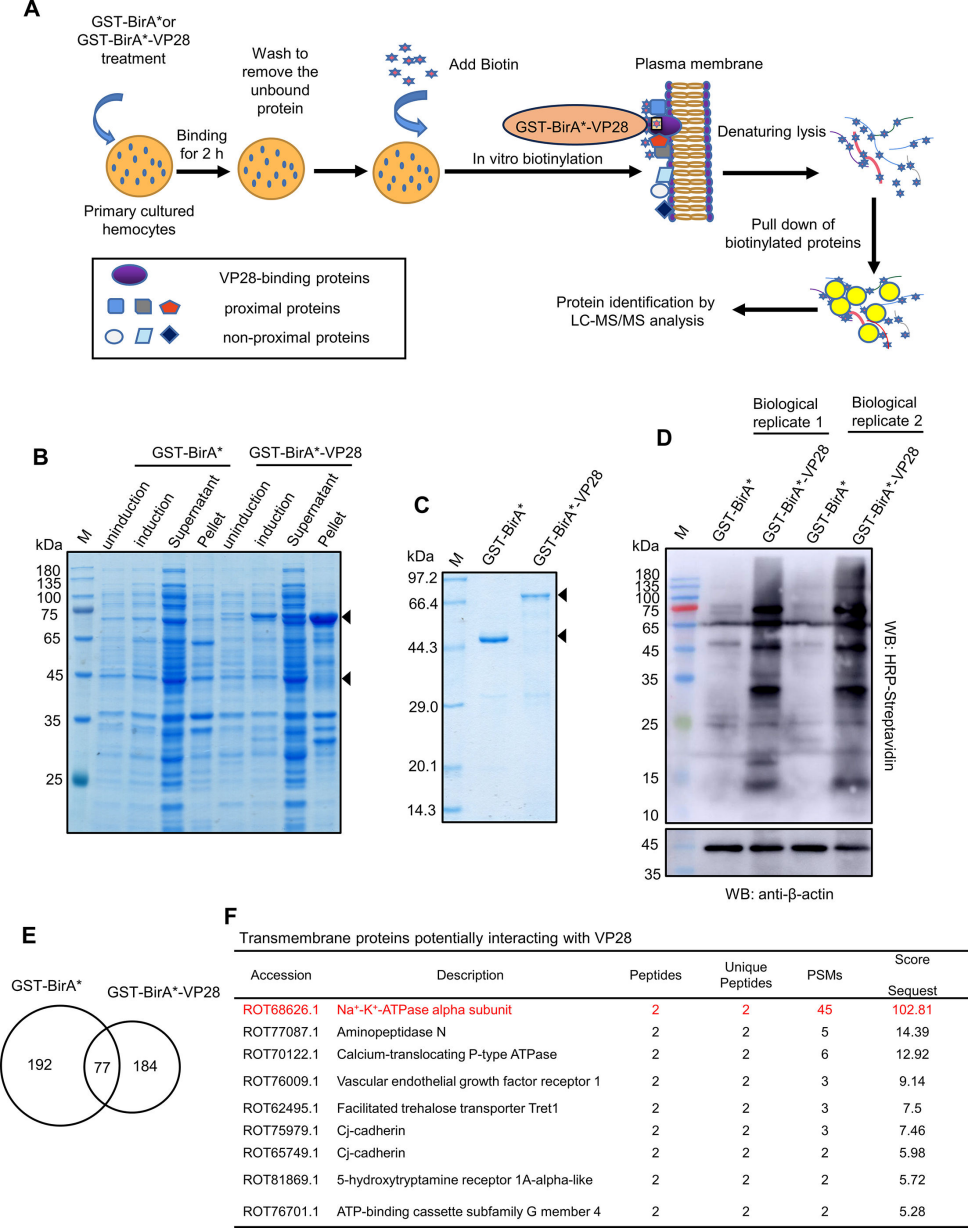
Identification of transmembrane proteins potentially interacting with WSSV VP28 protein by BioID screening. (**A**) Flow chart of the BioID screening approach. Recombinant proteins GST-BirA* and GST-BirA*-VP28 were incubated with primary cultured hemocytes for 2 h. Following washing to remove unbound proteins, the cells underwent *in vitro* biotinylation, followed by denaturing lysis, biotin pull-down, and LC-MS/MS analysis. (**B and C**) Expression and purification of the recombinant proteins GST-BirA* and GST-BirA*-VP28. These proteins were expressed in *Escherichia coli* BL21 cells (**B**) and purified using glutathione Sepharose 4B (**C**). M: protein marker. (**D**) Analysis of biotinylation activity of GST-BirA* and GST-BirA*-VP28 proteins. Hemocytes treated with GST-BirA* or GST-BirA*-VP28 were lysed and analyzed by a western blot using an HRP-streptavidin antibody. (**E**) Venn diagram showing the numbers of identified proteins in GST-BirA* and GST-BirA*-VP28 treatments by LC-MS/MS analysis. (**F**) Summary of transmembrane proteins that potentially interacted with VP28 protein. Proteins uniquely identified in the GST-BirA*-VP28 group were assessed using TMHMM-2.0 software, and those predicted to possess transmembrane helices were considered candidate virus receptors.

Given that the most abundant envelope protein of WSSV, VP28, acts as a viral attachment protein to facilitate viral infection (26, 27), this study used VP28 as a bait protein to screen for new potential cell surface receptors, including transmembrane proteins, using the BioID interaction proteomics technique (Fig. 1A). Biotinylated proteins were detected in hemocytes treated with both GST-BirA* and GST-BirA*-VP28, with a higher abundance observed in the latter group, indicating promiscuous biotinylation activity (Fig. 1B through D). When the biotinylated proteins in shrimp hemocytes were captured using NeutrAvidin Agarose Resins and analyzed by liquid chromatography-tandem mass spectrometry (LC-MS/MS), 269 proteins were identified in the GST-BirA* control group and 261 proteins in the GST-BirA*-VP28 group (Fig. 1E; Table S1). When proteins uniquely identified in the GST-BirA*-VP28 group are considered the VP28-interacting proteins, 184 candidate proteins were recognized, with nine predicted as transmembrane proteins, including Na^+^-K^+^-ATPase alpha subunit (ATP1A), aminopeptidase N (APN), and calcium-translocating P-type ATPase (PCMA) (Fig. 1F). ATP1A was selected for further investigation because it had the most number of secondary MS spectra and the highest MS score.

### Shrimp ubiquitously express *Pv*ATP1A that is induced by WSSV infection

**Fig 2:**
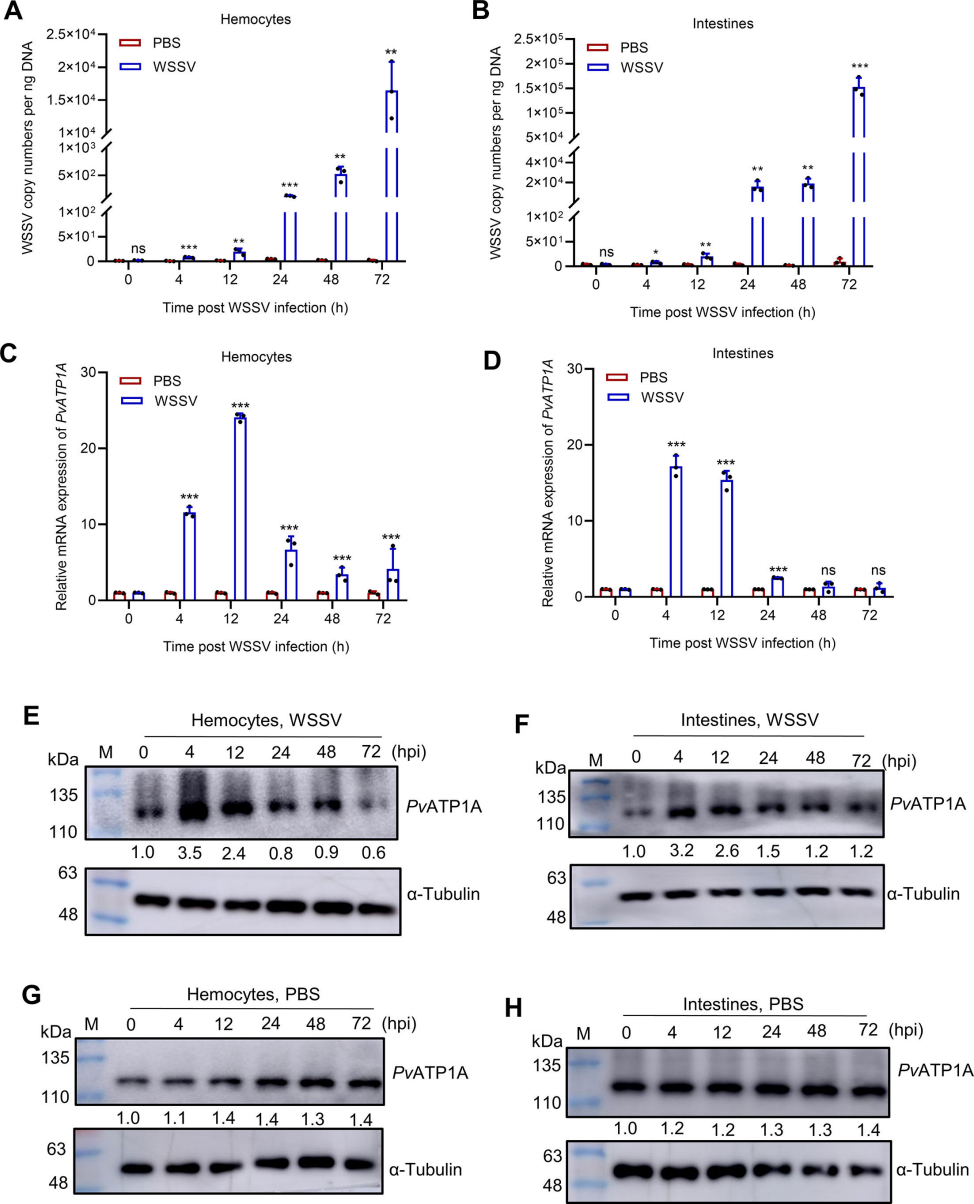
Upregulation of *Pv*ATP1A in WSSV-challenged shrimp. (**A–D**) Quantification of WSSV load and *Pv*ATP1A mRNA expression in hemocytes and intestines. Hemocytes and intestines were collected at 0, 4, 12, 24, 48, and 72 h post-WSSV or PBS injection for subsequent DNA and RNA extraction. Quantification of WSSV copy numbers (**A and B**) and *Pv*ATP1A mRNA expression (**C and D**) was performed using qPCR. *Pv*ATP1A mRNA expression of the PBS group at each time point was set to 1.0. (**E–H**) Detection of *Pv*ATP1A protein expression in hemocytes and intestines. The hemocytes and intestines collected at the indicated time points following WSSV (**E and F**) or PBS injection (**G and H**) were lysed for western blot analysis using an anti-ATP1A antibody. The gray values obtained from the western blot results were analyzed utilizing ImageJ software and subsequently normalized to the PBS group at 0 h. Statistical significance between groups was determined by two-tailed Student’s *t*-test. *P < 0.05, ***P < 0.01, and ***P < 0.001; ns: not significance.

The Na^+^-K^+^-ATPase alpha subunit homolog in *P. vannamei* (*Pv*ATP1A) was initially cloned and identified in 2015 (GenBank accession no. KF765670.1) with a full-length cDNA of 3,117 bp open reading frame encoding a 1,038-amino acid protein (28). This study investigated the tissue distribution of *Pv*ATP1A. Due to the unavailability of a specific *Pv*ATP1A antibody, a rabbit anti-ATP1A antibody from Abcam, generated against an immunogen derived from *Danio rerio* ATP1A (GenBank accession no. NP_571764, amino acids 620–868), was employed. This antibody exhibited specificity for *Pv*ATP1A in shrimp through Western blot analysis, supported by over 89% immunogenic amino acid sequence similarity with *Pv*ATP1A (Fig. S1A and B). We found that the mRNA transcripts and *Pv*ATP1A proteins were ubiquitously expressed across all examined shrimp tissues, with higher levels in the intestine, hemocytes, and nerve (Fig. S1C and D). Given the high expression of *Pv*ATP1A in the intestine and hemocytes, coupled with the fact that the intestine serves as the primary site of WSSV natural infection while hemocytes are essential for systemic infection (29, 30), the mRNA and protein expression of *Pv*ATP1 in hemocytes and intestines was analyzed at various time points post-WSSV or -PBS injection. Following WSSV injection, a progressive increase was observed in viral copy numbers in hemocytes and intestines post-WSSV injection compared with the PBS control, confirming successful WSSV infection (Fig. 2A and B). *Pv*ATP1A mRNA and protein expression showed an initial increase during the early phase (0–12 h) of WSSV infection, followed by a decline thereafter, from 24 to 72 h compared with the PBS control (Fig. 2C through H). The response of *Pv*ATP1A to WSSV challenge suggests its potential involvement in WSSV infection.

### *Pv*ATP1A promotes WSSV infection in penaeid shrimp

**Fig 3:**
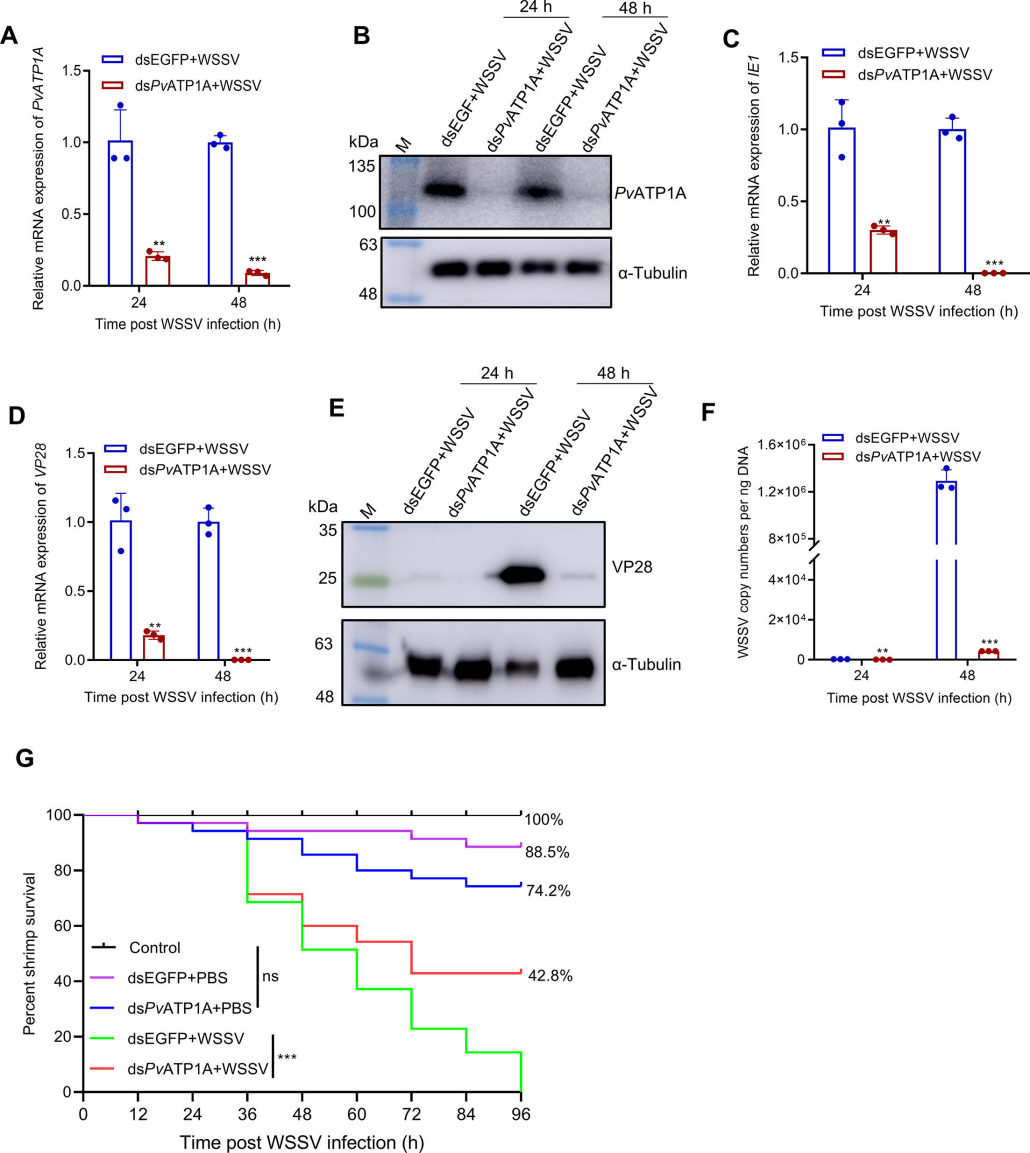
Suppression of WSSV infection in hemocytes following *Pv*ATP1A knockdown. (**A and B**) Knockdown efficiency analysis of *Pv*ATP1A in hemocytes. Shrimps were individually injected with dsEGFP and ds*Pv*ATP1A, then infected with WSSV at 48 h post-dsRNA injection. Hemocytes were harvested at 24 and 48 h post-viral infection to assess *Pv*ATP1A mRNA and protein levels using qPCR (**A**) and western blot (**B**) analyses. (**C–E**) Quantification of the mRNA and protein expression of WSSV genes. The mRNA levels of the WSSV genes *IE1* (**C**) and *VP28* (**D**) following *Pv*ATP1A knockdown were determined by qPCR, whereas the VP28 protein level was measured by western blot using a VP28 antibody. (**F**) Quantification of WSSV copy numbers following *Pv*ATP1A knockdown by qPCR. (**G**) Shrimp survival rate after *Pv*ATP1A knockdown followed by WSSV or PBS injection. Statistical significance was assessed using a two-tailed Student’s *t*-test for comparisons between two groups (**A**, **C**, **D**, and **F**) and the log-rank test in GraphPad Prism for survival rate analysis. *P < 0.05, ***P < 0.01, and ***P < 0.001.

To elucidate the role of *Pv*ATP1A in WSSV infection, an RNAi assay was conducted. After RNAi-mediated knockdown of *Pv*ATP1A followed by WSSV infection, significant reductions in *Pv*ATP1A mRNA and protein levels were observed in hemocytes (Fig. 3A and B). Similarly, the transcripts of WSSV genes, that is, *IE1* and *VP28,* and the viral protein VP28 expression were markedly decreased in *Pv*ATP1A-silenced hemocytes (Fig. 3C through E). Additionally, knockdown of *Pv*ATP1A also significantly attenuated WSSV viral copy numbers in hemocytes (Fig. 3F). Next, we examined the effect of *Pv*ATP1A knockdown on WSSV infection in the intestines. Similarly, the knockdown of *Pv*ATP1A in the intestines drastically reduced WSSV gene expression and viral copy numbers (Fig. S2A through E). Moreover, following WSSV infection, the knockdown of *Pv*ATP1A significantly enhanced shrimp survival rate compared with dsEGFP control (Fig. 3G). These results suggest that *Pv*ATP1A facilitates WSSV infection in penaeid shrimp.

### *Pv*ATP1A undergoes oligomerization, clustering, and internalization after WSSV infection

**Fig 4:**
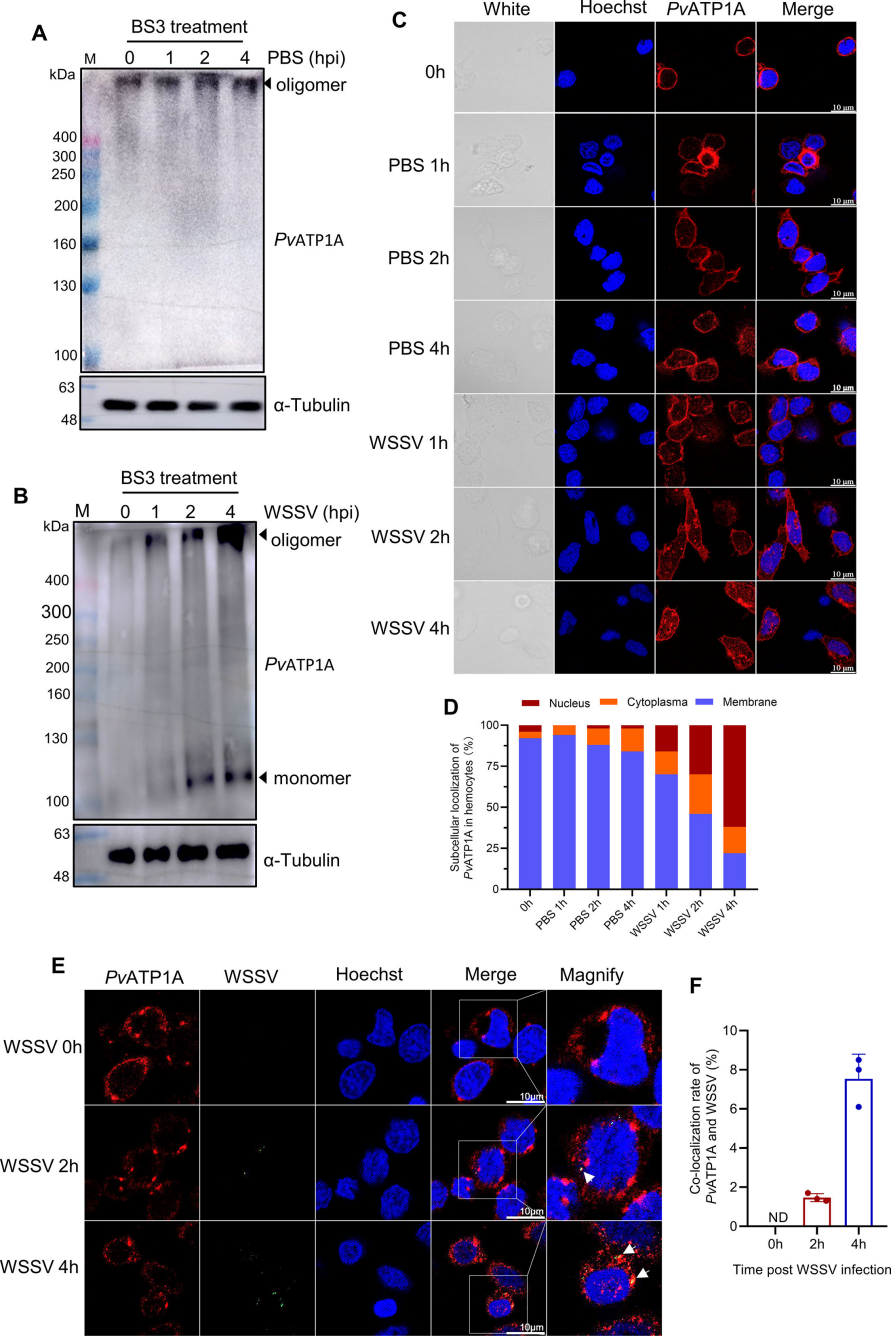
Oligomerization, clustering, and internalization of *Pv*ATP1A following WSSV infection. (**A and B**) Oligomerization analysis of *Pv*ATP1A. Hemocytes were collected at 0, 1, 2, and 4 h post-PBS (**A**) or WSSV (**B**) injection, treated with BS3, and analyzed by western blot analysis with an anti-ATP1A antibody. M: protein marker. (**C and D**) Subcellular localization analysis of *Pv*ATP1A. Hemocytes collected at 0, 1, 2, and 4 h after WSSV or PBS injection were subjected to immunofluorescence staining with an anti-ATP1A antibody (**B**). The percentage of *Pv*ATP1A subcellular localization in hemocytes was quantified in panel **C** with 50 hemocytes counted and analyzed per treatment. (**E and F**) Colocalization analysis of *Pv*ATP1A and WSSV particles. Hemocytes collected at 0, 2,nucleus and membrane. and 4 h post-infection were stained with anti-ATP1A and anti-VP28 antibodies. Representative immunofluorescence images are shown in panel **E**, and the colocalization rate is quantified in panel **F**. Data were from three independent experiments, with at least 200 hemocytes counted per replicate. ND: not detected.

To explore the mechanisms by which *Pv*ATP1A promotes WSSV infection, the subcellular localization of *Pv*ATP1A and any potential structural changes it undergoes after WSSV infection were determined. After PBS injection, *Pv*ATP1A was observed as an oligomer rather than a monomer in hemocytes, as evidenced by a protein with a larger molecular weight identified by the anti-ATP1A antibody (Fig. 4A). This suggests that *Pv*ATP1A functions as an oligomer under physiological conditions. When shrimp were infected with WSSV, both monomeric and oligomeric forms of *Pv*ATP1A were detected, with the levels of oligomerization increasing over the course of WSSV infection from 0 to 4 h (Fig. 4B), indicating that WSSV infection could promote *Pv*ATP1A oligomerization. Immunofluorescence staining with the anti-ATP1A antibody revealed a uniform distribution of *Pv*ATP1A on the plasma membrane in the PBS control group at all time points. In contrast, after WSSV infection, *Pv*ATP1A clustered and was internalized into the perinuclei and nuclei of hemocytes (Fig. 4C and D). For colocalization analysis, *Pv*ATP1A and WSSV particles were analyzed using immunofluorescence assay with anti-ATP1A and anti-VP28 antibodies, respectively. *Pv*ATP1A and WSSV were found to colocalize at 2 and 4 h post-infection (Fig. 4D). Moreover, the colocalization of *Pv*ATP1A and WSSV was time-dependent as the colocalization rate was 1.5% at 2 h, increasing to 7.5% at 4 h (Fig. 4E). These results suggest that *Pv*ATP1A plays a role in the early phase of WSSV infection, such as viral entry.

### *Pv*ATP1A facilitates WSSV entry

**Fig 5:**
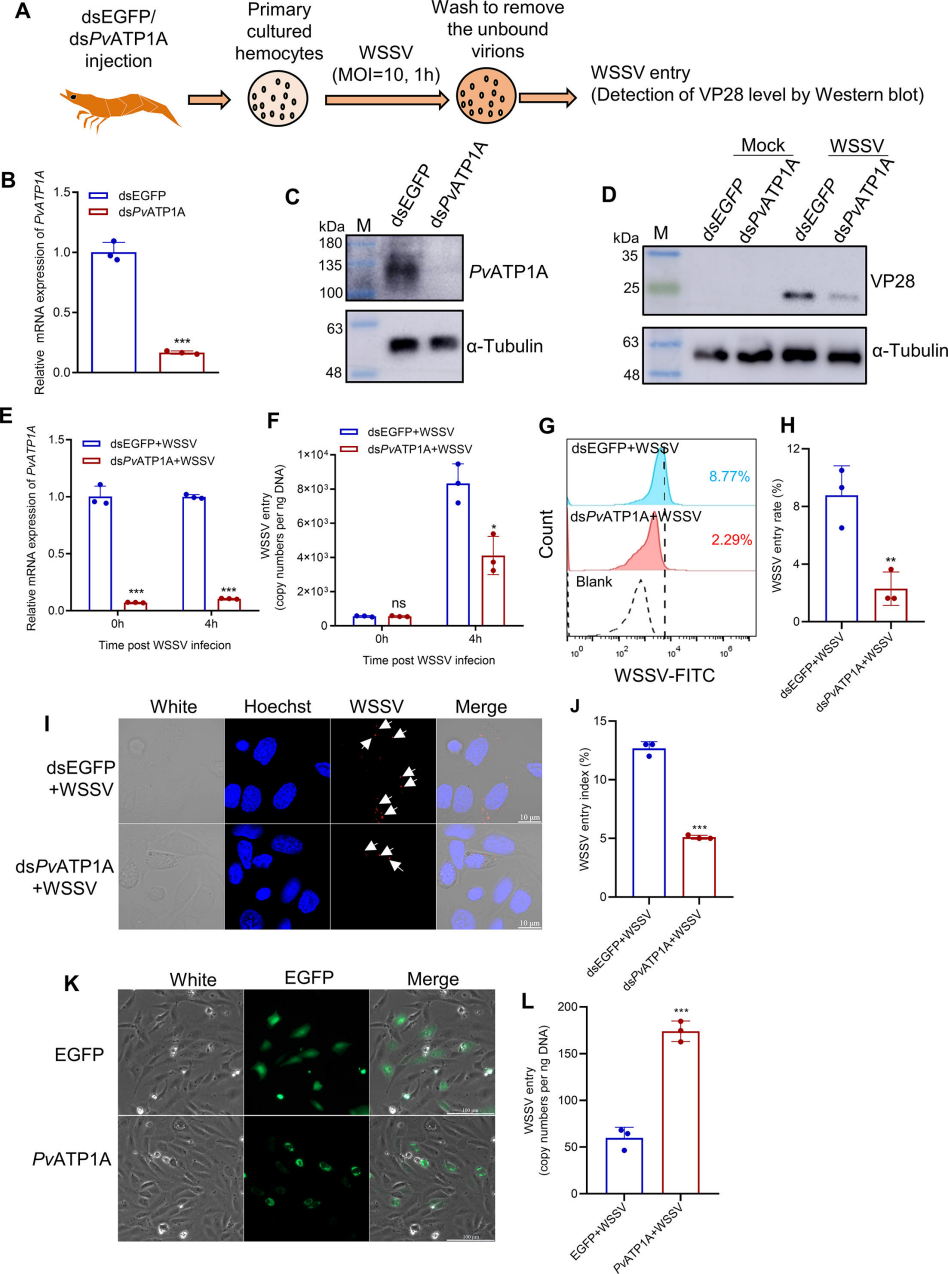
*Pv*ATP1A facilitates WSSV entry. (**A–D**) *In vitro* assessment of WSSV entry following *Pv*ATP1A knockdown. Hemocytes collected from shrimp before and after *Pv*ATP1A knockdown and cultured in insect-XPRESS medium were either mock-infected or exposure to WSSV, and virus entry was assessed by western blot (**A**). The efficiency of *Pv*ATP1A knockdown was confirmed via qPCR (**B**) and western blot (**C**), and virus entry was evaluated by Western blot using an anti-VP28 antibody (**D**). (**E–H**) *In vivo* analysis of WSSV entry following *Pv*ATP1A knockdown. FITC-labeled or unlabeled WSSV particles were injected into shrimp before and after *Pv*ATP1A knockdown. Hemocytes collected at 4 h post-infection were used to assess knockdown efficiency (**E**) and determine viral entry by qPCR (**F**) and flow cytometry (**G**). Entry rate was quantified in panel **H** based on data from three independent flow cytometry experiments. (**I and J**) Immunofluorescence evaluation of WSSV entry *in vivo*. Unlabeled WSSV particles were injected into shrimp before and after *Pv*ATP1A knockdown, and hemocytes were harvested at 4 h post-infection for immunofluorescence staining with an anti-VP28 antibody. Representative immunofluorescence images were shown in panel **I**, with the WSSV entry index quantified in panel **J**. Data were obtained from three independent experiments, with a minimum of three hundred hemocytes counted per replicate. (**K and L**) WSSV entry in non-permissive cells after *Pv*ATP1A overexpression. Zebrafish PAC2 fibroblast cells transfected with *Pv*ATP1A or EGFP expression plasmids (**K**) were infected with WSSV for 1 h. Following the removal of the uninfected virions, WSSV entry was assessed by qPCR (**L**). Statistical significance was determined using a two-tailed Student’s *t*-test. *P < 0.05, ***P < 0.01, and ***P < 0.001; ns: not significance.

To determine whether *Pv*ATP1A is essential for viral entry, we conducted RNAi-mediated knockdown and overexpression experiments. Hemocytes from *Pv*ATP1A-depleted or control shrimp were incubated *in vitro* with WSSV for 1 h, followed by washing off unbound viral particles and cell lysis. When VP28 levels in the cell lysates were determined by western blot as a measure of how much virions entered hemocytes (Fig. 5A), there was a significant reduction in VP28 levels in the *Pv*ATP1A-depleted hemocytes compared with control (Fig. 5B thorugh D), indicating a decrease in viral entry. Similarly, after *Pv*ATP1A *in vivo* knockdown followed by injection with fluorescein isothiocyanate (FITC)-labeled or unlabeled WSSV, qPCR and flow cytometry analysis of shrimp hemocytes at 4 h post-infection revealed a significant reduction in viral copy numbers and virus entry rate compared with control (Fig. 5E through H). Immunofluorescence analysis with an anti-VP28 antibody confirmed a substantial decrease in virus entry index in *Pv*ATP1A knockdown hemocytes compared with controls (Fig. 5I and J). When *Pv*ATP1A was overexpressed in non-permissive zebrafish PAC2 fibroblast cells to explore the effect of *Pv*ATP1A on WSSV entry (Fig. 5K), a significant increase in viral copy numbers was observed in the *Pv*ATP1A overexpressing cells compared with control (Fig. 5L).

Given that WSSV has been reported to enter shrimp tissues using other cell surface entry receptors, such as β-integrin, Glut1, and pIgR (11, 18, 21), we compared these receptors with *Pv*ATP1A. Shrimp were depleted of these entry receptors using RNAi-mediated knockdown followed by injection with either unlabeled or FITC-labeled WSSV, and hemocytes were collected at 4 h post-infection for analysis of WSSV entry using flow cytometry and qPCR. After achieving a knockdown efficiency of approximately 70% (Fig. S3A), we also observed a significant decrease in both virus entry rate and viral copy numbers in hemocytes compared with the control (Fig. S3B through D). Most importantly, the decrease in viral entry rate and copy numbers in hemocytes was most prominent in *Pv*ATP1A-silenced hemocytes compared with the other entry receptors (i.e., β-integrin, Glut1, and pIgR). These results indicate that compared with the other previously identified WSSV entry receptors, *Pv*ATP1A plays a more crucial role in WSSV infection.

### *Pv*ATP1A binds to WSSV VP28 protein via its multiple extracellular regions

**Fig 6:**
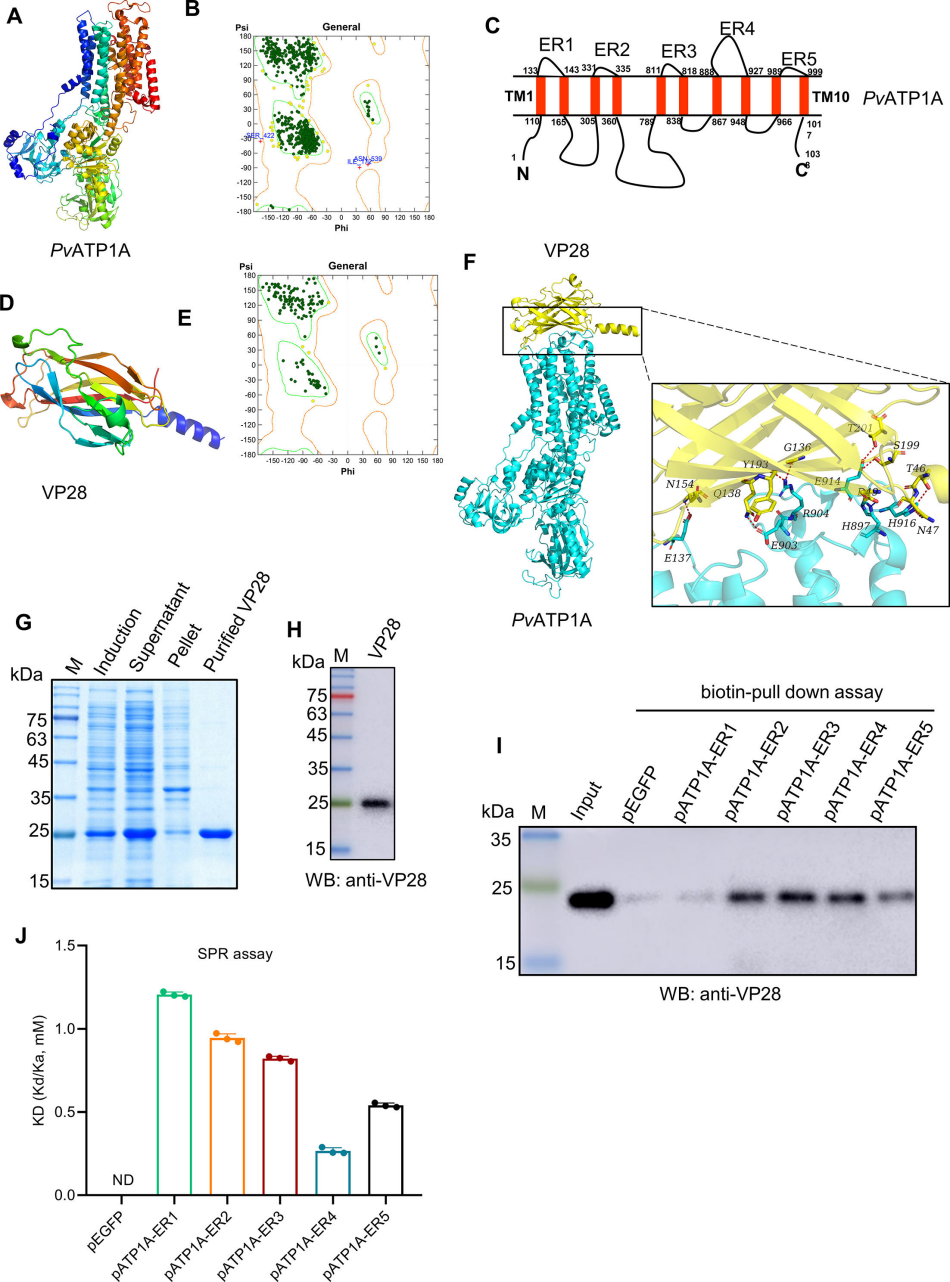
*Pv*ATP1A binds to WSSV VP28 protein via its multiple extracellular regions. (**A–C**) Homology modeling of *Pv*ATP1A. The 3D structure of *Pv*ATP1A was predicted using homology modeling (**A**) and validated by Ramachandran plot analysis (**B**), where dark green and yellow dots indicate residues in favored regions, respectively. The primary structure of *Pv*ATP1A determined from its 3D model is illustrated in panel **C**. (**D and E**) Crystal structure of WSSV VP28. The VP28 structure was obtained from the RCSB Protein Data Bank (**D**) and evaluated by Ramachandran plot analysis (**E**). (**F**) Molecular docking of *Pv*ATP1A and VP28. *Pv*ATP1A (cyan) and VP28 (yellow) are shown with their residues represented as sticks, whereas hydrogen bonds are indicated by red dashes and salt bridges by blue dashes. (**G–I**) Interaction analysis of *Pv*ATP1A and VP28 via biotin pull-down. Recombinant VP28 protein was expressed and purified from *E. coli* BL21 cells (**G**) and confirmed by western blot with an anti-VP28 antibody (**H**). The purified VP28 was incubated with five biotin-labeled synthetic peptides corresponding to the extracellular regions of *Pv*ATP1A, with a synthetic EGFP peptide (pEGFP) as a negative control (**I**). M: protein marker. (**J**) Binding affinity between *Pv*ATP1A extracellular region peptides and VP28 as determined by SPR analysis. ND indicates not detected.

When the interaction between *Pv*ATP1A and VP28 was investigated using molecular docking, the three-dimensional (3D) structure of *Pv*ATP1A was first predicted through homology modeling, utilizing the crystal structure of *Squalus acanthias* ATP1A (PDB No. 2ZXE) as the reference template, with a sequence identity of 74.08%. After generating the *Pv*ATP1A model (Fig. 6A), the analysis of the Ramachandran plot revealed that more than 99% of the residues in *Pv*ATP1A were within the permitted areas, confirming the model’s validity (Fig. 6B). Next, homology modeling revealed that *Pv*ATP1A is a 10-transmembrane protein with the N- and C-terminals located on the cytoplasmic side, consisting of five extracellular regions (ER1–ER5) and 10 transmembrane regions (TM1–TM10) (Fig. 6C). When the crystal structure of VP28 (PDB No. 2ED6), obtained from the Research Collaboratory for Structural Bioinformatics (RCSB) Protein Data Bank (Fig. 6D and E), was used for molecular docking analysis with *Pv*ATP1A employing HDOCK2, the results revealed that the residues Glu137, His897, Glu903, Arg904, Glu914, and His916 in *Pv*ATP1A interacted with residues Thr46, Asn47, Asp49, Gly136, Gln138, Asn154, Tyr193, Ser199, and Thr201 in VP28 via salt bridges and hydrogen bonds (Fig. 6F; Table S2). Notably, Glu137 is situated in the ER1 of *Pv*ATP1A, whereas the remaining four residues are located in ER4, indicating that VP28 binds to both the first and fourth extracellular regions of *Pv*ATP1A.

To confirm the interaction between *Pv*ATP1A and VP28, five biotin-labeled synthetic peptides of the extracellular regions of *Pv*ATP1A (designated pATP1A-ER1 to pATP1-ER5) were used in a biotin pulldown assay with recombinant VP28 protein (Fig. 6G and H). All the five *Pv*ATP1A extracellular synthetic peptides could bind to VP28 (Fig. 6I). When surface plasmon resonance (SPR) tests were used to determine their binding affinity using the purified recombinant VP28 immobilized onto a CM5 sensor chip and different concentrations of the synthetic peptides, it was found that the flow-through of the control peptide pEGFP caused no changes in the response units (RU) of the VP28-coated chip, but the RU increased in a concentration-dependent manner for the five *Pv*ATP1A extracellular peptides, with the fourth peptide (pATP1A-ER4) causing the greatest increase (Fig. S4 A through F). When the equilibrium dissociation constant (KD) for the interactions between the *Pv*ATP1A extracellular region peptides and VP28 was determined, the KD values were 0 mM for pEGFP, 1.21 ± 0.015 mM for pATP1A-ER1, 0.944 ± 0.025 mM for pATP1A-ER2, 0.821 ± 0.014 mM for pATP1A-ER3, 0.266 ± 0.019 mM for pATP1A-ER4, and 0.541 ± 0.0136 mM for pATP1A-ER5 (Fig. 6J). Taken together, these results suggest that all five *Pv*ATP1A extracellular region peptides can bind to VP28, with peptide pATP1A-ER4 showing the highest affinity.

### Synthetic *Pv*ATP1A extracellular region peptides block WSSV infection

**Fig 7:**
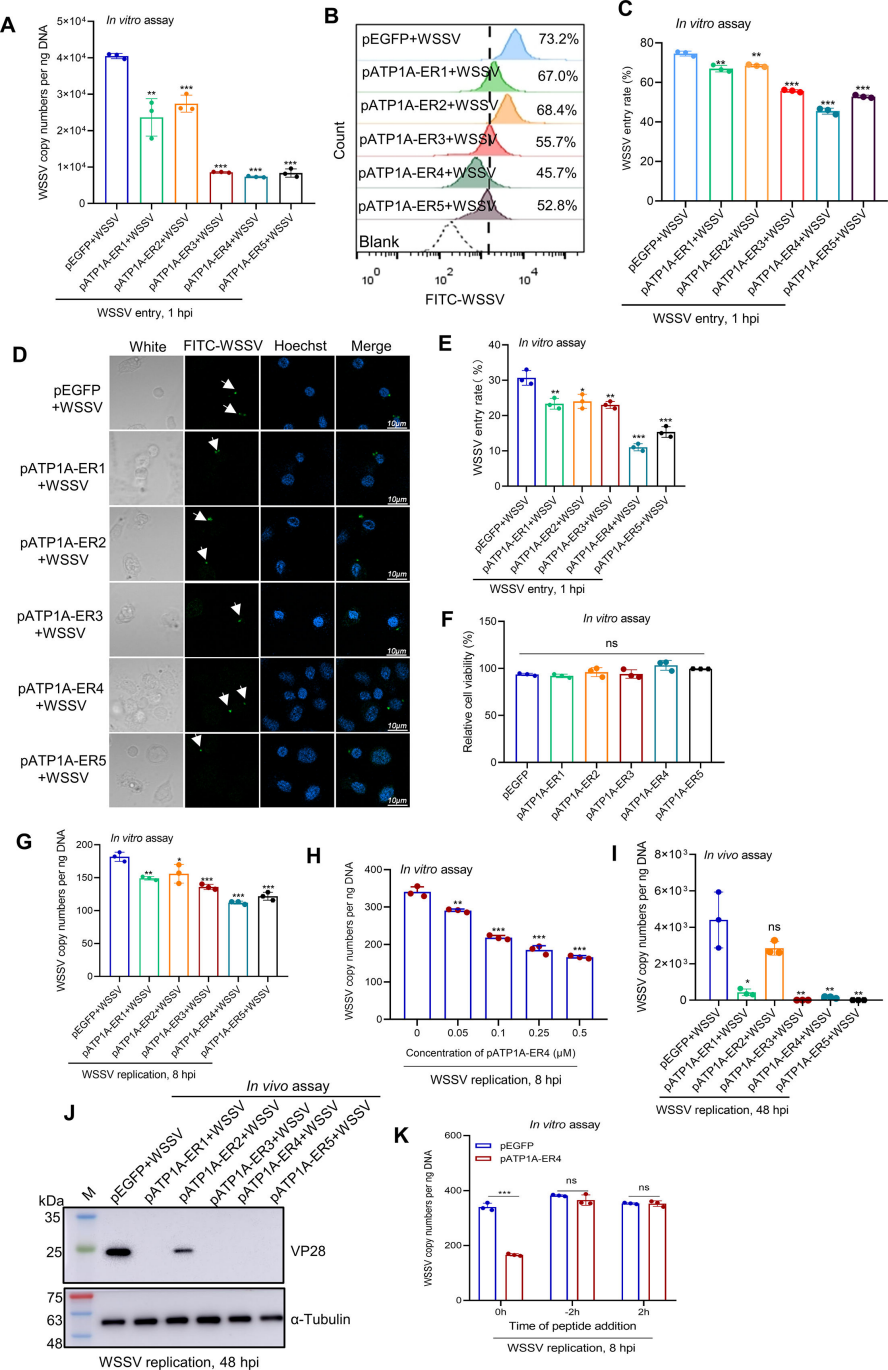
Synthetic *Pv*ATP1A extracellular region peptides inhibit WSSV infection. (**A–E**) Impact of peptide blocking on WSSV entry. WSSV particles were pre-incubated with synthetic peptides from the extracellular regions of *Pv*ATP1A or a control peptide (pEGFP) for 2 h, followed by infection of primary cultured hemocytes for 1 h. Virus entry was assessed by qPCR (**A**), flow cytometry (**B**), and immunofluorescence (**D**). Data from three independent experiments are summarized in panels **C** and **E** for flow cytometry and immunofluorescence, respectively. (**F–J**) Effect of peptide blocking on WSSV replication. Hemocyte viability post-treatment with synthetic *Pv*ATP1A extracellular peptides was measured using the CCK-8 assay (**F**). WSSV particles, pre-treated with synthetic *Pv*ATP1A peptides (pATP1A-ER1-5) or varying concentrations of pATP1A-ER4, were used to infect hemocytes *in vitro* or injected into shrimp *in vivo*. After 8 h of *in vitro* infection or 48 h *in vivo*, hemocytes were collected to quantify WSSV copy numbers and VP28 levels by qPCR (**G–I**) and western blot (**J**). (**K**) Time-of-peptide-addition analysis with pATP1A-ER4. Hemocytes were treated concurrently with pATP1A-ER4 and WSSV, pre-treated with pATP1A-ER4 prior to WSSV infection, or infected with WSSV before pATP1A-ER4 treatment. After infection for 8 h, hemocytes were collected for DNA extraction, and WSSV copy numbers were quantified by qPCR. Statistical significance was determined using a two-tailed Student’s *t*-test. *P < 0.05, **P < 0.01, and ***P < 0.001; ns, not significant.

To explore whether the binding between *Pv*ATP1A and VP28 would influence WSSV entry, peptide-blocking assays were performed. Unlabeled or FITC-labeled WSSV particles were pre-treated with synthetic *Pv*ATP1A extracellular region peptides or a control peptide pEGFP *in vitro* for 2 h, followed by infection of primary cultured hemocytes and analyzed using qPCR, flow cytometry, and immunofluorescence assay to determine viral entry. Significant decreases were observed in the viral copy numbers, virus entry rate, and virus entry index in the *Pv*ATP1A extracellular peptides treated group compared with the pEGFP control (Fig. 7A through E). Notably, peptide pATP1A-ER4 exhibited the most potent inhibitory effect on WSSV entry.

To investigate whether synthetic *Pv*ATP1A extracellular peptides could inhibit WSSV replication post-entry blockade, primary cultured hemocytes were infected with WSSV particles that had been pre-treated with the synthetic *Pv*ATP1A extracellular peptides or a control peptide pEGFP, for 2 h *in vitro*. When WSSV replication was assessed by qPCR after 8 h of *in vitro* infection, all five synthetic *Pv*ATP1A extracellular region peptides, especially peptide pATP1A-ER4, could significantly reduce viral copy numbers without causing cytotoxicity to hemocytes compared with the control peptide (Fig. 7F and G). When the dose-dependent inhibitory effect of peptide pATP1A-ER4 on WSSV replication was further examined, we observed a concentration-dependent suppression of WSSV replication (Fig. 7H). To further examine the impact of the five synthetic *Pv*ATP1A extracellular region peptides on WSSV replication *in vivo*, shrimp were injected with WSSV particles pre-incubated with the synthetic *Pv*ATP1A extracellular region peptides or the control peptide pEGFP for 2 h. When WSSV replication was analyzed using qPCR and western blot analyses at 48 h post-infection using hemocytes, the five *Pv*ATP1A extracellular region peptides significantly reduced viral copy numbers and VP28 protein expression compared with the control peptide (Fig. 7I and J). To confirm that the inhibition of WSSV replication by the extracellular region peptides of *Pv*ATP1A is due to suppression of viral entry, a time-of-peptide-addition experiment was performed using peptide pATP1A-ER4. Primary cultured hemocytes were concurrently treated with pATP1A-ER4 and WSSV, or pre-treated with pATP1A-ER4 for 2 h before WSSV infection, or infected with WSSV for 2 h before being treated with pATP1A-ER4. When viral replication was examined on these three treatment conditions, it was found that peptide pATP1A-ER4 could only inhibit WSSV replication when co-administered with the virus (Fig. 7K). These results indicate that peptide pATP1A-ER4 inhibits the entry of WSSV to attenuate viral infection.

### *Pv*ATP1A facilitates WSSV internalization via multiple endocytic pathways

**Fig 8:**
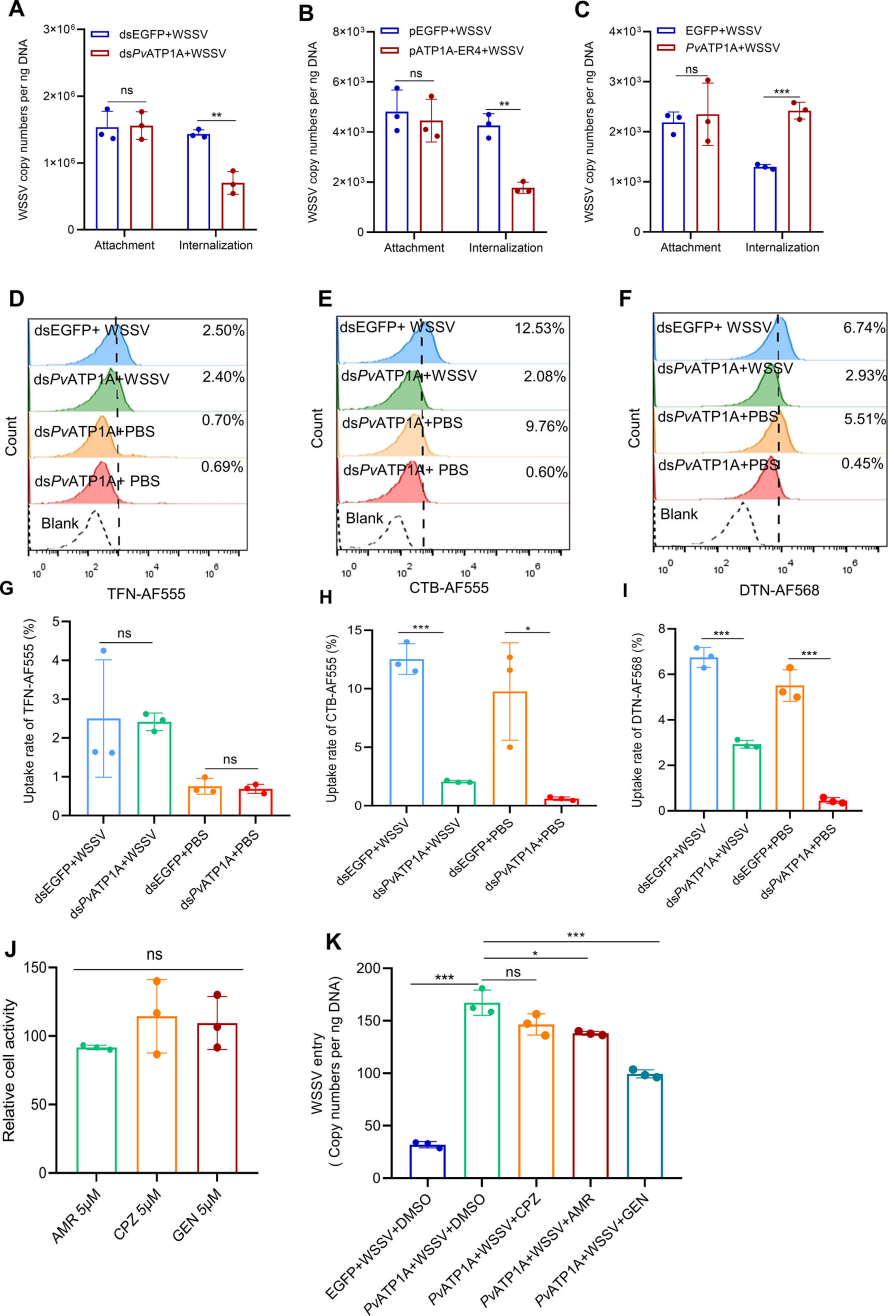
*Pv*ATP1A facilitates WSSV internalization via caveolin-mediated endocytosis and macropinocytosis. (**A–C**) Role of *Pv*ATP1A in WSSV attachment and internalization. WSSV particles were used to infect shrimp hemocytes and zebrafish PAC2 fibroblast cells before and after *Pv*ATP1A knockdown (**A**) or overexpression (**C**), respectively. Viral attachment and internalization were assessed by qPCR. Additionally, WSSV particles pre-treated with synthetic peptides (pATP1A-ER or pEGFP) were used to analyze viral attachment and internalization in hemocytes (**B**). (**D–I**) Impact of *Pv*ATP1A on endocytic marker uptake. Hemocytes from shrimp pre- and post-*Pv*ATP1A knockdown were incubated with endocytic markers (TFN-AF555, CTB-AF555, and DTN-AF568) in the presence of WSSV or PBS. After 1 h of infection, uptake rates were evaluated by flow cytometry. Representative images for TFN-AF555, CTB-AF555, and DTN-AF568 uptake are shown in panels **D**, **E**, and **F** with quantification provided in panels **G**, **H**, and **I** from three independent replicates. (**J–K**) Effect of endocytic inhibitors on WSSV entry in *Pv*ATP1A-overexpressing cells. Zebrafish PAC2 cells treated with inhibitors (AMR, CPZ, and GEN) were evaluated for cell viability using the CCK8 assay (**J**). Subsequently, cells overexpressing *Pv*ATP1A were pre-treated with these inhibitors or DMSO (negative control) before WSSV infection. After 1 h of infection, the cells were rinsed and WSSV copy numbers were quantified by qPCR (**K**). Statistical significance was determined using a two-tailed Student’s *t*-test. *P < 0.05, **P < 0.01, and ***P < 0.001; ns, not significant.

Given that viruses initiate their entry process by adhering to the host cell’s surface to be internalized into cells, we explored the precise step of WSSV entry affected by *Pv*ATP1A. Hemocytes collected from shrimp pre- and post-*Pv*ATP1A knockdown were infected with WSSV. Subsequent viral attachment and internalization analysis showed no decrease in attached virions but a significant reduction in internalized virions in *Pv*ATP1A-silenced hemocytes (Fig. 8A). Similarly, there was no change in viral attachment, but a significant decrease in internalized virions was observed when the primary cultured hemocytes were infected with WSSV pre-treated with peptide pATP1A-ER4 compared with the control (Fig. 8B). We also demonstrated using non-permissive cells that the overexpression of *Pv*ATP1A had no impact on WSSV attachment, although it significantly enhanced viral internalization (Fig. 8C). These results indicate that *Pv*ATP1A does not affect viral attachment but internalization during WSSV entry.

Given that viruses enter host cells by exploiting various endocytic pathways, such as clathrin-mediated endocytosis, caveolin-mediated endocytosis, and macropinocytosis (31), we went on to examine which of these endocytic pathways is used by WSSV to enter hemocytes. Shrimp were injected with either WSSV or PBS, and hemocytes were collected after 4 h. These hemocytes were then incubated with Alexa Fluor-conjugated transferrin (TFN-AF555), cholera toxin subunit B (CTB-AF555), and dextran (DTN-AF568) to track the internalization process via clathrin-mediated endocytosis, caveolin-mediated endocytosis, and macropinocytosis, respectively (32–34). The flow cytometry results showed that the uptake rates of TFN-AF555, CTB-AF555, and DTN-AF568 were all significantly increased in WSSV-infected hemocytes (Fig. S5 A through F), indicating the activation of all three endocytic pathways after WSSV infection. When shrimp were injected with various endocytic inhibitors, that is, chlorpromazine hydrochloride (CPZ), a clathrin-mediated endocytosis inhibitor; genistein (GEN), a caveolin-mediated endocytosis inhibitor; and amiloride hydrochloride (AMR), a macropinocytosis inhibitor before being infected with WSSV and analyzed using qPCR and flow cytometry, all the endocytic inhibitors inhibited WSSV entry and replication, with GEN being the most potent inhibitors of virus entry (Fig. S5G through K). These findings suggest that WSSV enters shrimp hemocytes using multiple endocytic pathways, especially caveolin-mediated endocytosis.

To further investigate the specific endocytic pathway affected by *Pv*ATP1A in facilitating WSSV internalization, hemocytes collected from shrimp before and after *Pv*ATP1A knockdown were incubated *in vitro* with endocytic markers and with either WSSV or PBS for 1 h. When these hemocytes were washed with PBS before being used to determine the uptake rates of TFN-AF555, CTB-AF555, and DTN-AF568 using flow cytometry, there was a significant decrease in the uptake rates of CTB-AF555 and DTN-AF568 after *Pv*ATP1A knockdown, whereas that in the TFN-AF555-treated hemocytes remained unchanged (Fig. 8D through I). *Pv*ATP1A was overexpressed in zebrafish fibroblast cells, followed by treatment with endocytic inhibitors to examine the effect on WSSV entry. Treatment with the inhibitors at a concentration of 5 µM had no impact on cell viability (Fig. 8J). Cell overexpressing *Pv*ATP1A had significantly increased viral copy numbers compared with untransfected cells (Fig. 8K). Moreover, treatment of *Pv*ATP1A-overexpressing cells with the endocytic inhibitors GEN and AMR significantly decreased viral copy numbers compared with cells treated with CPZ (Fig. 8K). Collectively, these results suggest that *Pv*ATP1A facilitates WSSV internalization via caveolin-mediated endocytosis and macropinocytosis.

## DISCUSSION

WSSV has a broad host tropism across decapod crustaceans, which is capable of infecting various tissues and cell types, including the epidermis, gill, intestine, antennal gland, lymphoid organ, muscle, eyestalk, heart, gonads, hemocytes, hematopoietic cells, and nervous system-associated cells (9, 30). The diverse range of cells infected by WSSV indicates that it uses multiple cell surface receptors to target and infect different cell types, tissues, and species. Currently, although several receptors have been implicated in WSSV infection, such as Rab7, CBP, LamR, CTL, Bip, β-integrin, Glut1, and pIgR (35), most of which mainly serve as attachment factors rather than entry receptors (21). The inability to identify many WSSV entry receptors could be due to the limitations of the methods used. For this reason, the current study employed the BioID method, which has two major advantages over previous methods, that is, biotin labeling that enables the detection of transient and weak protein interactions, and the purification of the labeled products using biotin’s high affinity for streptavidin magnetic beads, allowing for cell lysis under denaturing conditions, which is particularly suitable for transmembrane protein identification. Nine transmembrane proteins, such as ATP1A, APN, and PCMA, that interacted with WSSV envelope protein VP28 and were potentially involved in WSSV infection were identified, whereas ATP1A, which had the highest protein score, was further characterized.

ATP1A is the major subunit of Na^+^-K^+^-ATPase, a transmembrane complex that transports sodium and potassium ions across the plasma membrane to maintain intracellular electrolyte and fluid balance (36, 37). This complex also includes a β subunit and an γ subunit (also referred to as the FXYD protein). ATP1A is an integral membrane protein with 10 transmembrane helices that form an ion channel, whereas the β and γ subunits are single transmembrane proteins that modulate the ion transport capabilities of Na^+^-K^+^-ATPase and contribute to the stability of the complex (37). In humans, ATP1A has four isoforms encoded by the *ATP1A*1-4 genes and shares 78%–87% sequence similarity. These isoforms exhibit diverse expression patterns across cell types, with ATP1A1 being ubiquitously expressed in tissues. In shrimp, a single *ATP1A* gene has thus far been identified in the *P. vannamei* genome (38). Here, we found that the *P. vannamei* homolog of ATP1A (*Pv*ATP1A) was expressed in all examined shrimp tissues (i.e., hemocyte, intestine, nerve, muscle, stomach, hepatopancreas, heart, and gill). Recent studies have shown that ATP1A1 is a pro-viral factor with distinct roles in the infection cycles of various viruses. For instance, ATP1A1 is essential for the cell entry of coronavirus, respiratory syncytial virus (RSV), and Porcine epidemic diarrhea virus (PEDV) (39–41). Conversely, chemical inhibition of ATP1A with drugs, such ouabain and digoxin, suppresses the replication of Zika virus (ZIKV), Chikungunya virus (CHIKV), and lymphocytic choriomeningitis virus (LCMV) at post-entry stages (42–44). In the current study, we found that WSSV infection induced the mRNA and protein expression of *Pv*ATP1A in the early infection stages and that RNAi-mediated knockdown of *Pv*ATP1A reduced WSSV infection and enhanced shrimp survival, suggesting that *Pv*ATP1A also plays a pro-viral role in WSSV infection.

To explore the mechanism by which *Pv*ATP1A enhances WSSV infection, we examined the oligomerization and subcellular localization of *Pv*ATP1A following WSSV infection. We found that WSSV infection promoted *Pv*ATP1A oligomerization and clustering at the early infection stages, a characteristic feature also observed in ATP1A1, a receptor for RSV (40). Given that oligomerization and clustering are features found in signaling receptors when they bind ligands (45, 46), it is possible that *Pv*ATP1A is potentially involved in initiating cellular signaling cascades in response to WSSV infection. At the early infection stages, *Pv*ATP1A was also internalized into hemocytes and colocalized with WSSV particles. This could be due to the fact that five synthetic extracellular region peptides of *Pv*ATP1A interacted with the viral envelope protein VP28. This observation is similar to previous findings, where the binding mechanism between the neuropilin 1 (NRP1) receptor and reovirus depended on multiple extracellular domains of NRP1 (47). This multisite binding likely induces receptor clustering, potentially activating signaling pathways and/or facilitating the recruitment to endocytic structures. Therefore, given the oligomerization, clustering, and internalization of *Pv*ATP1A and its colocalization with WSSV, *Pv*ATP1A might be crucial in the initial stages of WSSV infection, such as viral entry. Indeed, RNAi-mediated knockdown, and overexpression of *Pv*ATP1A, followed by WSSV infection revealed that *Pv*ATP1A knockdown attenuated WSSV entry into shrimp hemocytes, whereas the overexpression of *Pv*ATP1A in non-permissive cells enhanced virus entry. When compared with other previously identified receptors, such as β-integrin, Glut1, and pIgR (11, 18, 21), *Pv*ATP1A was found to be essential for WSSV internalization but not viral attachment, and it also enhanced viral entry much more than the other receptors. Therefore, *Pv*ATP1A is a crucial entry receptor for WSSV.

Most enveloped viruses use receptor-mediated endocytic mechanisms, like clathrin-mediated endocytosis, caveolin-mediated endocytosis, and macropinocytosis to enter host cells (31). Previous studies have shown that WSSV enters host cells, such as hematopoietic tissue (HPT) cells of the red claw crayfish, *Cherax quadricarinatus*, through various endocytic pathways (48, 49). However, in other shrimp cells, such as hemocytes, the specific endocytic pathways used by WSSV have not been fully elucidated. The current study shows that WSSV infection activates various endocytic processes, including clathrin-mediated endocytosis, caveolin-mediated endocytosis, and macropinocytosis, as evidenced by the increased uptake rates of endocytic markers after WSSV infection. In shrimp, inhibition of these pathways using various endocytic inhibitors, that is, CPZ, GEN, and AMR, significantly reduced WSSV entry and replication, suggesting that WSSV utilizes multiple endocytic pathways in hemocytes. Interestingly, several recent studies have revealed that ATP1A regulates these endocytic pathways. For example, ATP1A functions as a signaling anchor to regulate the localization and activation of phosphoinositide-3 kinase (PI3K), which is essential for the binding of adaptor protein 2 and recruitment of clathrin (50). ATP1A also interacts with caveolin-1 to regulate caveolin-1 endocytic trafficking (51). In addition, ATP1A activates Src-EGFR signaling to induce cytoskeletal rearrangement that facilitates RSV entry through macropinocytosis (40). Our study showed that RNAi-mediated knockdown of *Pv*ATP1A inhibited caveolin-mediated endocytosis and macropinocytosis, but not clathrin-mediated endocytosis. Moreover, in *Pv*ATP1A overexpressing cells, treatment with inhibitors of caveolin-mediated endocytosis, and macropinocytosis attenuated WSSV entry. In contrast, treatment with an inhibitor of clathrin-mediated endocytosis did not impact viral entry. These findings indicate that *Pv*ATP1A promotes WSSV internalization through caveolin-mediated endocytosis and macropinocytosis.

**Fig 9:**
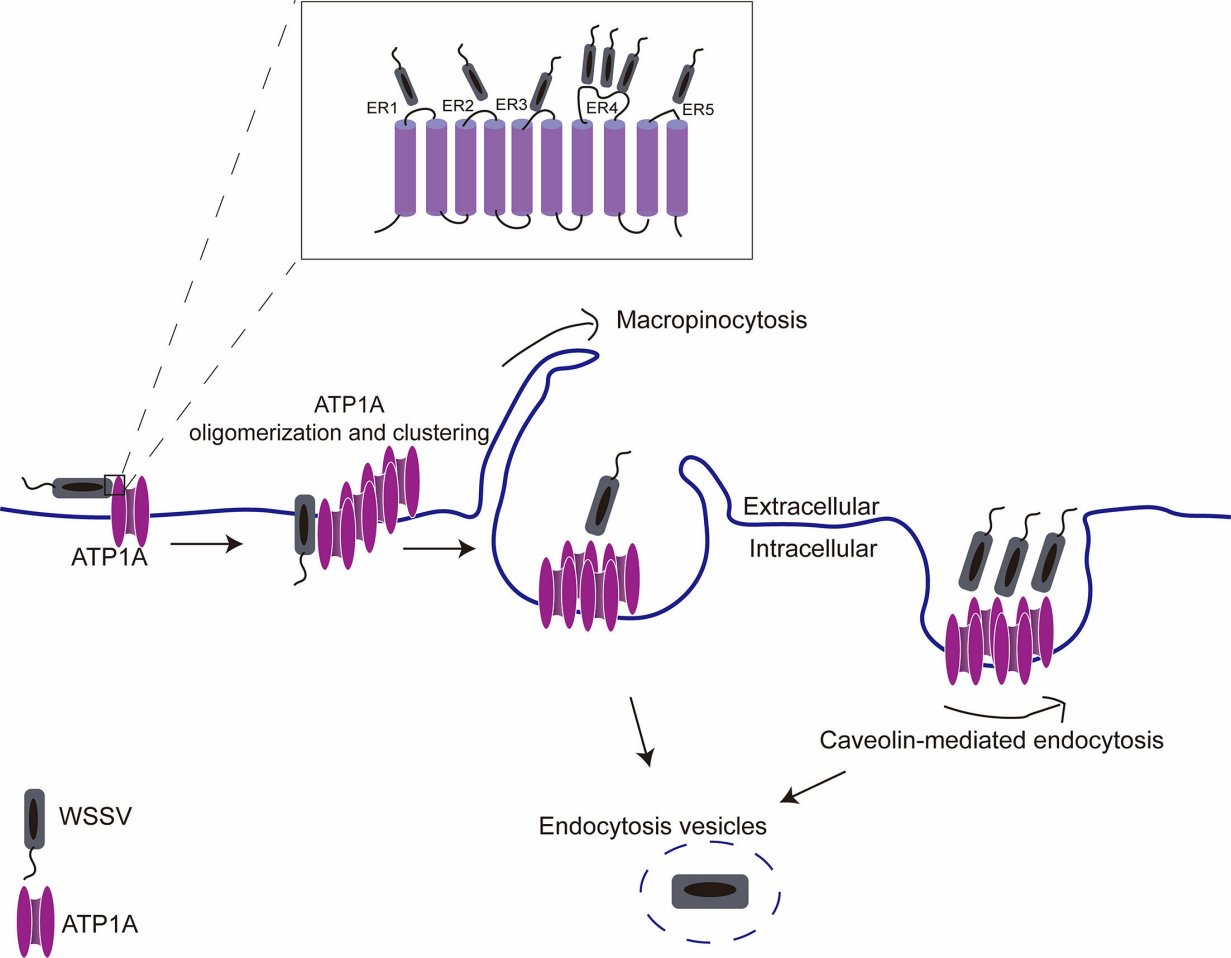
Schematic representation of *Pv*ATP1A mediating WSSV entry in shrimp. This study illustrates that during the early stages of WSSV infection; *Pv*ATP1A was upregulated and underwent oligomerization, clustering, and internalization. *Pv*ATP1A interacts with the WSSV envelope protein VP28 through multiple extracellular regions, facilitating viral internalization via caveolin-mediated endocytosis and macropinocytosis.

In conclusion, our data demonstrate that during the early stages of WSSV infection, *Pv*ATP1A is induced, followed by its oligomerization, clustering, and internalization. Most importantly, *Pv*ATP1A interacts with the WSSV envelope protein VP28 through its multiple extracellular regions to facilitate viral internalization via caveolin-mediated endocytosis and macropinocytosis (Fig. 9). The findings here provide a more robust receptor screening approach, which helped us identify for the first time, *Pv*ATP1A as an entry receptor for WSSV in shrimp.

## MATERIALS AND METHODS

### Experimental animals

Healthy *P. vannamei* (5–8 g) was sourced from a local shrimp farm in Shantou, Guangdong Province, China. The shrimp were acclimated in laboratory tanks with air-circulated artificial seawater (salinity: 5 ‰, temperature: 25°C) for a minimum of 2 days prior to experimentation and were fed commercial diets once daily.

### Antibodies and plasmids

The primary antibodies employed in this study included HRP-streptavidin (Genscript, China, cat. no. M00091), mouse monoclonal anti-α-tubulin (Sigma-Aldrich, USA, cat. No. T6074), rabbit anti-β-actin (Beyotime, China, Cat. No. AF5003), and rabbit polyclonal anti-ATP1A (Abcam, UK, Cat. No. ab211130). The mouse monoclonal anti-VP28 antibody was generously provided by Prof. Feng Yang from the Third Institute of Oceanography, Ministry of Natural Resources (Xiamen, China). For western blotting, secondary antibodies included goat anti-mouse IgG-HRP (ThermoFisher Scientific, USA, Cat. No. G21040) and goat anti-rabbit IgG-HRP (ThermoFisher Scientific, USA, Cat. No. G21234). For immunofluorescence, donkey anti-rabbit IgG-Alexa Fluor 555 (Beyotime, China, Cat. No. A0453) and goat anti-mouse IgG-Alexa Fluor 488 (Beyotime, China, Cat. No. A0428) were used.

The biotin ligase BirA* expression plasmid pGEX-BirA* was constructed by cloning the BirA* gene from the MCS-BioID2-HA vector (Addgene, USA, Cat. No. 74224) into the pGEX-6p-1 plasmid at the *Bam*H I restriction site using the ClonExpress Ultra One Step Cloning Kit (Vazyme, China, Cat. No. C116-01). The pGEX-BirA*-VP28 plasmid, expressing BirA*-fused VP28, was derived from pGEX-BirA* using the same cloning kit. The primers used for plasmid construction are listed in Table S3. The pEGFP-N1-*Pv*ATP1A plasmid was commercially synthesized based on the pEGFP-N1 vector. Additionally, the VP28 expression plasmid pET-His-VP28 was a kind gift from Prof. Feng Yang. Primers used for plasmid construction are listed in Table S2.

### BioID assay

Due to the lack of shrimp cell lines and considering hemocytes are essential for systemic infection of WSSV (30), we chose primary cultured hemocytes for the BioID experiment. Hemocytes were obtained from shrimp using sterile syringes containing an anticoagulant buffer (140 mM NaCl, 32 mM citric acid, 29 mM sodium citrate, pH 6.0). Subsequently, the hemocytes were pelleted by centrifugation at 600 × *g* for 10 min at 4°C and cultured in Insect-XPRESS medium (LONZA, Switzerland, Cat. No. 12-730Q) at 28°C. The BioID assay was performed following a previously established protocol with minor modifications (52). Briefly, plasmids pGEX-GST-BirA* and pGEX-GST-BirA*-VP28 were transformed into *E. coli* BL21 cells to produce and purify recombinant GST-BirA* and GST-BirA*-VP28 proteins. These proteins were incubated separately with hemocytes. After a 2 h binding period, hemocytes were washed with phosphate-buffered saline (PBS) (140 mM NaCl, 2 mM NaH_2_PO4, 10 mM Na_2_HPO_4_, pH 7.4) to remove unbound proteins. The cells were then treated with a biotinylation buffer (50 µM biotin, 40 mM Tris-HCl, 5 mM MgCl2, 3 mM ATP, 100 µM KCl, pH 8.0) for *in vitro* biotin labeling. Following biotinylation, the cells were harvested, and lysed, and a portion of the lysate was analyzed by western blot using HRP-streptavidin to assess promiscuous biotinylation activity of GST-BirA* and GST-BirA*-VP28. The remaining lysate was subjected to biotin pull-down using NeutrAvidin Agarose Resins (ThermoFisher Scientific, USA, Cat. No. 29202), and protein identification was carried out via LC-MS/MS analysis at the Medical College, Shantou University (Shantou, China).

### WSSV challenge, tissue collection, RNA, DNA, and protein extraction

The WSSV stock (China isolate) was obtained from WSSV-infected crayfish (*Procambarus clarkii*) and quantified by qPCR as described previously (53, 54). Each shrimp was injected with 100 µL containing 1 × 10^5^ copies of WSSV, whereas the control group received an equivalent volume of sterile PBS. Hemocytes were collected from the shrimp at 0, 4, 12, 24, 48, and 72 h post-infection using sterile syringes with an anticoagulant buffer and pelleted by centrifugation at 600 × *g* for 10 min at 4°C. Intestinal tissues were dissected on ice using scissors and forceps. Total RNA was extracted from hemocytes and intestines using an RNA rapid extraction kit (Fastagen, China, Cat. No. 220010) and reverse transcribed into cDNA with a cDNA synthesis kit (TransGen Biotech, China, Cat. No. AT311-02) for qPCR analysis. Genomic DNA was extracted with the TIANamp Marine Animals DNA Kit (TIANGEN, China, Cat. No. DP324) and quantified by qPCR to determine viral copy numbers after WSSV infection as described previously (54). For protein extraction, hemocytes and intestines were homogenized in RIPA buffer (50 mM Tris-HCl, 150 mM NaCl, 0.1% SDS, 0.5% Nonidet P-40, 1 mM EDTA, 0.5 mM PMSF, pH 7.5). The homogenates were centrifuged at 20,000 × *g* for 10 min at 4°C, and the supernatants were used for western blot analysis.

### qPCR and western blot assay

The mRNA expression levels of *PvATP1A* following WSSV infection were quantified using qPCR. The reaction mixture included 5 µL of 2× RealStar Green Power Mix (GenStar, Beijing, China, cat. no. A311), 1 µL each of forward and reverse primers, 1 µL of cDNA template, and 3 µL of Milli-Q water. qPCR was performed on a LightCycler 480 (Roche, Switzerland) with the following conditions: initial denaturation at 95°C for 10 min, followed by 45 cycles of 95°C for 15 s and 60°C for 30 s. The mRNA expression of *PvATP1A*, normalized to elongation factor 1-α (*Pv*EF-1α), was calculated using the 2^−ΔΔCT^ method. Primers for *PvATP1A* and *Pv*EF-1α are listed in Table S3. For protein expression analysis of *Pv*ATP1A post-WSSV infection, western blotting was performed. Protein samples were resolved on 10% SDS-PAGE gels and transferred to polyvinylidene fluoride (PVDF) membranes (Millipore, USA, Cat. No. IPVH00010). Membranes were blocked with 5% (wt/vol) skim milk in TBST buffer (20 mM Tris, 150 mM NaCl, 0.1% Tween 20, pH 7.6) at room temperature for 1 h. They were then incubated with primary antibodies (anti-ATP1A, 1:2,000; anti-α-tubulin, 1:3,000) for 2 h, followed by HRP-conjugated secondary antibodies (1:3,000) for 1 h at room temperature. Protein bands were visualized using Immobilon Forte Western HRP Substrate (Millipore, USA, Cat. No. WBLUF0100) and captured with the Amersham Imager 600 (GE Healthcare, USA).

### RNAi assay

The dsRNA targeting *PvATP1A* (ds*Pv*ATP1A) was synthesized *in vitro* using the HiScribe T7 Quick High Yield RNA Synthesis Kit (New England Biolabs, USA, Cat. No. E2050S) according to the manufacturer’s protocol. As a negative control, dsRNA targeting EGFP (dsEGFP) was also synthesized. Shrimps were randomly assigned to two groups, each receiving an intramuscular injection of 100 µL containing 15 µg of either ds*Pv*ATP1A or dsEGFP. At 48 h post-dsRNA injection, each shrimp was further injected with 100 µL of 1 × 10^5^ copies of WSSV. Hemocytes and intestines were harvested at 24 and 48 h post-infection for RNA, DNA, and protein extraction. RNA and protein samples were analyzed to evaluate the knockdown efficiency of *PvATP1A* and the expression of WSSV genes (i.e., *IE1* and *VP28*) using qPCR and western blot. Genomic DNA was extracted using the TIANamp Marine Animals DNA Kit and quantified via qPCR to assess viral copy numbers pre- and post-PvATP1A knockdown in hemocytes and intestines. Primers used in this study are listed in Table S3.

### Survival rate assay

Shrimp were intramuscularly injected with 100 µL of 15 µg of ds*Pv*ATP1A or dsEGFP. Following a 48 h RNAi period, each group (*n* = 35) was subdivided into two subgroups. One subgroup from each original group was further injected with 100 µL containing 1 × 10^5^ copies of WSSV, whereas the other subgroup received an equal volume of sterile PBS. Additionally, shrimps without any treatments were cultured and employed as a negative control. Mortality was monitored at 12 h intervals post-virus infection. Survival curves were generated, and statistical significance was evaluated using the log-rank test with GraphPad Prism software.

### Oligomerization assay

The oligomerization of *Pv*ATP1A following WSSV infection was assessed with minor modifications to a previously described method (21). Shrimp were intramuscularly injected with 100 µL containing 1 × 10^5^ copies of WSSV. Hemocytes were collected at 0, 1, 2, and 4 h post-infection and washed three times with ice-cold PBS (20 mM sodium phosphate, 150 mM NaCl, pH 8.0) to remove amine-containing proteins. The hemocytes were then resuspended in PBS and crosslinked with 3-sulfo-N-hydroxysuccinimide ester (BS3; ThermoFisher Scientific, USA, Cat. No. 21586) at a final concentration of 5 mM. After a 1 h incubation at 4°C, crosslinking was quenched with 20 mM Tris-HCl (pH 7.5) for 30 min at room temperature. The hemocytes were subsequently lysed with SDS-PAGE sample loading buffer and analyzed by western blot using an anti-ATP1A antibody.

### Subcellular localization assay

The subcellular localization of *Pv*ATP1A after WSSV infection was analyzed using immunofluorescence. Shrimp were intramuscularly injected with 100 µL of WSSV (1 × 10^5^ copies) or PBS. Hemolymph was collected from three randomly selected shrimp at 0, 1, 2, and 4 h post-infection and centrifuged at 600 × *g* for 10 min at 4°C to isolate hemocytes. The hemocytes were resuspended in Insect-XPRESS medium, and 100 µL of the suspension (1 × 10^5^ cells) were plated in confocal dishes and cultured at 28°C for 1 h. Cells were then fixed with 4% paraformaldehyde for 15 min, permeabilized with 0.5% Triton X-100 in PBS for 20 min at room temperature, and washed three times with PBS. Blocking was performed with 3% bovine serum albumin (BSA) in PBS for 1 h at room temperature. The cells were incubated overnight at 4°C with anti-ATP1A antibody (1:200) diluted in 3% BSA. After three PBS washes, the cells were treated with donkey anti-rabbit IgG-Alexa Fluor 555 (1:400) diluted in 3% BSA for 1 h at room temperature. Following three additional washes with PBS, nuclei were stained with Hoechst 33342 (Beyotime, Shanghai, China, Cat. No. C10122). The slides were then examined using a laser confocal microscope (Carl Zeiss, Germany).

### Colocalization of *Pv*ATP1A and WSSV

Shrimp were intramuscularly injected with 100 µL of WSSV (1 × 10^8^ copies). Hemocytes were collected at 0, 2, and 4 h post-infection. The cells were subjected to immunofluorescence staining using anti-ATP1A and anti-VP28 antibodies as previously described. Briefly, after fixation, permeabilization, and blocking, hemocytes were incubated with anti-ATP1A and anti-VP28 antibodies (1:200, diluted in 3% BSA). This was followed by incubation with donkey anti-rabbit IgG-Alexa Fluor 555 and goat anti-mouse IgG-Alexa Fluor 488 (1:400, diluted in 3% BSA). After PBS washes, nuclei were stained with Hoechst 33342. Colocalization of *Pv*ATP1A and WSSV was assessed using a confocal laser scanning microscope. The colocalization rate was calculated as [number of hemocytes showing colocalization/total observed hemocytes] × 100%.

### Viral entry assay

The impact of *Pv*ATP1A knockdown on WSSV entry was evaluated using western blot, qPCR, flow cytometry, and immunofluorescence techniques. Hemocytes were isolated from shrimp before and after *Pv*ATP1A knockdown and cultured in Insect-XPRESS medium at 28°C. Cells were either mock-infected or infected with WSSV (MOI = 10) *in vitro*. After 1 h of infection, unbound viruses were removed by PBS washing, and cells were lysed for western blot analysis using anti-VP28 antibody to assess viral entry. For qPCR and flow cytometry, WSSV particles were fluorescently labeled with FITC as previously described (55). Shrimp pre- and post-*Pv*ATP1A knockdown were injected with either unlabeled or FITC-labeled WSSV particles (1 × 10^9^ copies). Hemocytes were collected at 4 h post-injection. A portion of the cells was used for DNA extraction to quantify WSSV copy numbers via qPCR, whereas the remainder was analyzed by flow cytometry to determine the WSSV entry rate. The entry rate was defined as (number of WSSV−positive hemocytes/total hemocytes analyzed) × 100%. For immunofluorescence, shrimp were injected intramuscularly with WSSV (1 × 10^8^ copies) before and after *Pv*ATP1A knockdown. Hemocytes were collected 4 h post-injection and subjected to immunofluorescence staining with anti-VP28 antibody to evaluate the viral entry index. The entry index was calculated as (number of virions in all hemocytes/total hemocytes observed ) × 100%.

### Overexpression of *Pv*ATP1A in WSSV nonpermissive cells

Zebrafish PAC2 fibroblast cells, kindly provided by Prof. Fan Lin from Shantou University (Shantou, China), were maintained at 28°C in L15 medium (Solarbio, China, Cat. No. LA9510) supplemented with 10% fetal bovine serum (Bio-channel, China, Cat. No. BC-SE-FBS01) and 1% penicillin-streptomycin (ThermoFisher Scientific, USA, Cat. No. 15140122). For DNA transfection, PAC2 cells (2 × 10^6^ cells/well) were seeded in a 24-well plate and transfected with either pEGFP-N1-*Pv*ATP1A or pEGFP-N1 (negative control) plasmids using FuGENE HD Transfection Reagent (Promega, USA, Cat. No. E2311) according to the manufacturer’s protocol. Forty-eight hours post-transfection, the cells were infected with WSSV (MOI = 0.1) for 1 h at 28°C. After infection, the cells were washed with PBS to remove unbound viral particles and then harvested for DNA extraction. The WSSV copy number was quantified using qPCR.

### Molecular docking assay

The molecular docking study of *Pv*ATP1A and VP28 was carried out by Wecomput Company in Beijing, China. To initiate the study, the *Pv*ATP1A sequence (GenBank accession no. KF765670.1) was used to identify a suitable template protein with high homology via the BLAST program, which led to the selection of the crystal structure of *Squalus acanthias* ATP1A (PDB No. 2ZXE) as the template. Homology modeling was performed using Molecular Operating Environment (MOE) software to construct the 3D structure of *Pv*ATP1A. The crystal structure of the VP28 protein was obtained from the RCSB Protein Data Bank. Docking of *Pv*ATP1A with VP28 was conducted using the HDOCK server, which employs a hybrid docking strategy to predict protein-protein binding complexes. In this docking process, *Pv*ATP1A served as the receptor and VP28 as the ligand. The docked structures and interface residues were analyzed using the MOE contact module, and molecular graphics were visualized with PyMOL.

### Biotin pull-down assay

The plasmid pET-His-VP28 was transformed into *E. coli* BL21 cells, and protein expression was induced with IPTG at 18°C. Following induction, the cells were harvested and sonicated in PBS on ice for 30 min. The supernatant was used to purify VP28 protein using Ni-NTA agarose beads according to the manufacturer’s protocol. The purity and identity of the VP28 protein were confirmed by Coomassie staining and Western blot analysis with an anti-VP28 antibody. For the biotin pull-down experiment, five biotin-labeled *Pv*ATP1A extracellular region peptides (pATP1A-ER1-5) and a control peptide (pEGFP) were synthesized by Genscript Biotechnology Company (Nanjing, China). The peptides were dissolved in dimethyl sulfoxide (DMSO), with their sequences provided in Table S4. A 4 µL aliquot of each synthetic peptide solution (1 µM) was individually incubated with 200 µL of VP28 (100 ng/µL) for 1 h at 4°C. Following incubation, NeutrAvidin Agarose Resins were added to the peptide-protein mixture and incubated for an additional 30 min at 4°C. The resins were then washed three times with PBS containing 1% Tween-20, and the protein was eluted with 1× SDS PAGE loading buffer. The protein samples were analyzed by western blot using an anti-VP28 antibody.

### SPR assay

SPR experiments were conducted using a Biacore T200 system (GE Healthcare, USA). The VP28 protein was purified and immobilized on a Series S CM5 sensor chip (Cytiva, USA, Cat. No. 29149603) using amine-coupling chemistry, achieving an immobilization level of 10,000–11,000 response units (RU). Synthetic *Pv*ATP1A extracellular region peptides and the control peptide pEGFP were prepared at final concentrations ranging from 0.0123 mM to 1 mM and flowed over the chip surface. Regeneration of the sensor chip was performed by injecting glycine (pH 3.0) for 30 s at a flow rate of 30 µL/min. The Biacore Insight Evaluation Software application was utilized to analyze the association and dissociation rates using a steady state affinity analysis, and the equilibrium dissociation constant (KD) was determined as the ratio of the dissociation rate to the association rate.

### Peptide blocking assay

The role of *Pv*ATP1A in WSSV infection was assessed using a peptide-blocking assay (55). Unlabeled and FITC-labeled WSSV particles (1 × 10^5^ copies) were pre-treated with 80 µM synthetic *Pv*ATP1A extracellular region peptides or a control peptide, pEGFP, for 2 h *in vitro*. These pre-treated viral particles were then used to infect primary cultured hemocytes at MOI values of 10, 30, and 100 for subsequent qPCR, flow cytometry, and immunofluorescence analyses, respectively. After a 1 h infection period, unbound virus particles were removed by PBS washing. Hemocytes were then collected for viral entry analysis via qPCR, flow cytometry, and immunofluorescence. For the viral replication assay *in vitro*, WSSV particles were pre-treated with 0.5 µM synthetic *Pv*ATP1A extracellular peptides (pATP1A-ER1-5) or varying concentrations of the fourth peptide, pATP1A-ER4, with pEGFP as the negative control, for 2 h *in vitro*. These pre-treated particles were used to infect primary cultured hemocytes (MOI = 1). After 8 h of infection, hemocytes were harvested, and DNA was extracted for quantification of WSSV copy numbers via qPCR. To investigate the *in vivo* impact of synthetic *Pv*ATP1A extracellular peptides on WSSV replication, WSSV particles (1 × 10^5^ copies) were pre-treated with 80 µM synthetic *Pv*ATP1A extracellular peptides or the control peptide, pEGFP, for 2 h *in vitro*. These particles were then individually injected into shrimp. At 48 h post-infection, hemocytes were harvested for DNA and protein extraction, followed by the assessment of WSSV replication using qPCR and western blot analysis.

### Time-of-peptide-addition assay

Hemocytes were isolated from shrimp hemolymph and cultured in Insect-XPRESS medium at 28°C. For the peptide-blocking assay, WSSV particles were pre-treated with 0.5 µM synthetic pATP1A-ER4 peptide for 2 h *in vitro* before being used to infect primary cultured hemocytes (MOI = 1). In parallel experiments, primary cultured hemocytes were pre-treated with pATP1A-ER4 peptide for 2 h prior to WSSV infection or were infected with WSSV for 2 h before being treated with pATP1A-ER4. After an 8 h infection period, the hemocytes were harvested, and DNA was extracted for quantifying WSSV copy numbers through qPCR analysis.

### Virus attachment and internalization assay

The viral attachment and internalization assays were conducted following a previously established protocol with minor modifications (56). Briefly, hemocytes from shrimp, both pre- and post-*Pv*ATP1A knockdown, and zebrafish PAC2 fibroblast cells, before and after *Pv*ATP1A overexpression, were infected with WSSV at a multiplicity of infection (MOI) of 100 for hemocytes and 1 for PAC2 cells, respectively. The infection was carried out for 1 h at 4°C. Additionally, hemocytes were infected with WSSV pretreated with either pATP1A-ER4 or pEGFP peptide and incubated at 4°C for 1 h. For the viral attachment assay, cells were washed twice with ice-cold PBS to remove unbound viruses and then collected for DNA extraction. Quantification of WSSV copy numbers was performed using qPCR. In the viral internalization assay, the infected cells were cultured in pre-warmed Insect-XPRESS medium and incubated at 28°C for 1 h to facilitate virus internalization. To remove surface-bound virions, cells were treated with 0.2 M glycine (pH 3.0) for 2 min at room temperature. After two additional PBS washes, cells were harvested for DNA extraction, and WSSV copy numbers were quantified by qPCR.

### Endocytic markers uptake assay

Endocytic markers, including Alexa Fluor-conjugated transferrin (TFN-AF555, Cat. No. C34776), cholera toxin subunit B (CTB-AF555, Cat. No. T35352), and dextran (DTN-AF568, Cat. No. D22912), were obtained from ThermoFisher Scientific. Hemocytes, isolated from shrimp before and after *Pv*ATP1A knockdown, were cultured in Insect-XPRESS medium at 28°C. One hour later, the cells were washed with PBS and resuspended in a fresh medium containing 10 µg/mL of each endocytic marker, either in the presence of WSSV (1 × 10^6^ copies) or PBS. Following 1 h incubation, hemocytes were washed three times with PBS and resuspended in PBS. Uptake rates were analyzed using a BD Accuri C6 Plus flow cytometer (BD Biosciences, USA).

### Endocytic inhibitor treatment assay

Endocytic inhibitors, chlorpromazine hydrochloride (CPZ, Cat. No. HY-B0407A), genistein (GEN, Cat. No. HY-14596), and amiloride hydrochloride (AMR, Cat. No. HY-B0285A), were purchased from MedChemexpress. Zebrafish PAC2 fibroblast cells were transfected with either the *Pv*ATP1A expression plasmid (pEGFP-N1-*Pv*ATP1A) or the control plasmid pEGFP-N1. Forty-eight hours post-transfection, cells expressing pEGFP-*Pv*ATP1A were pre-treated with 5 µM of each endocytic inhibitor (CPZ, GEN, and AMR) or DMSO (as a control) for 30 min. Following pre-treatment, cells were infected with WSSV (MOI = 0.1) for 1 h. The cells were then washed thrice with PBS, and DNA was extracted for quantification of WSSV copy numbers using qPCR. Cytotoxicity of the endocytic inhibitors on PAC2 cells was assessed using the CCK8 kit (Beyotime, China, Cat. No. C0037), following the manufacturer’s instructions.

### Statistical analysis

All values or percentages were obtained from three independent experiments. The data were presented as means and standard deviations, with statistical significance determined using a two-tailed Student’s *t*-test. All figures were graphed and analyzed using GraphPad Prism software.

## Data Availability

All data are available in the main text or the supplemental materials.

## SUPPLEMENTAL MATERIAL

Supplemental figures and tables mbio.03787-24-s0001.docx Figures S1 to S5; Tables S1 to S4.

ASM does not own the copyrights to Supplemental Material that may be linked to, or accessed through, an article. The authors have granted ASM a non-exclusive, world-wide license to publish the Supplemental Material files. Please contact the corresponding author directly for reuse.
