# Supplementary material for: The Na+-K+-ATPase alpha subunit is an entry receptor for white spot syndrome virus
Source: mBio. 2025 Feb 18;16(3):e03787-24. doi: 10.1128/mbio.03787-24 (PMC11898654; doi:10.1128/mbio.03787-24)
Supplement: Supplemental figures and tables — Figures S1 to S5; Tables S1 to S4. [file mbio.03787-24-s0001.docx]

**
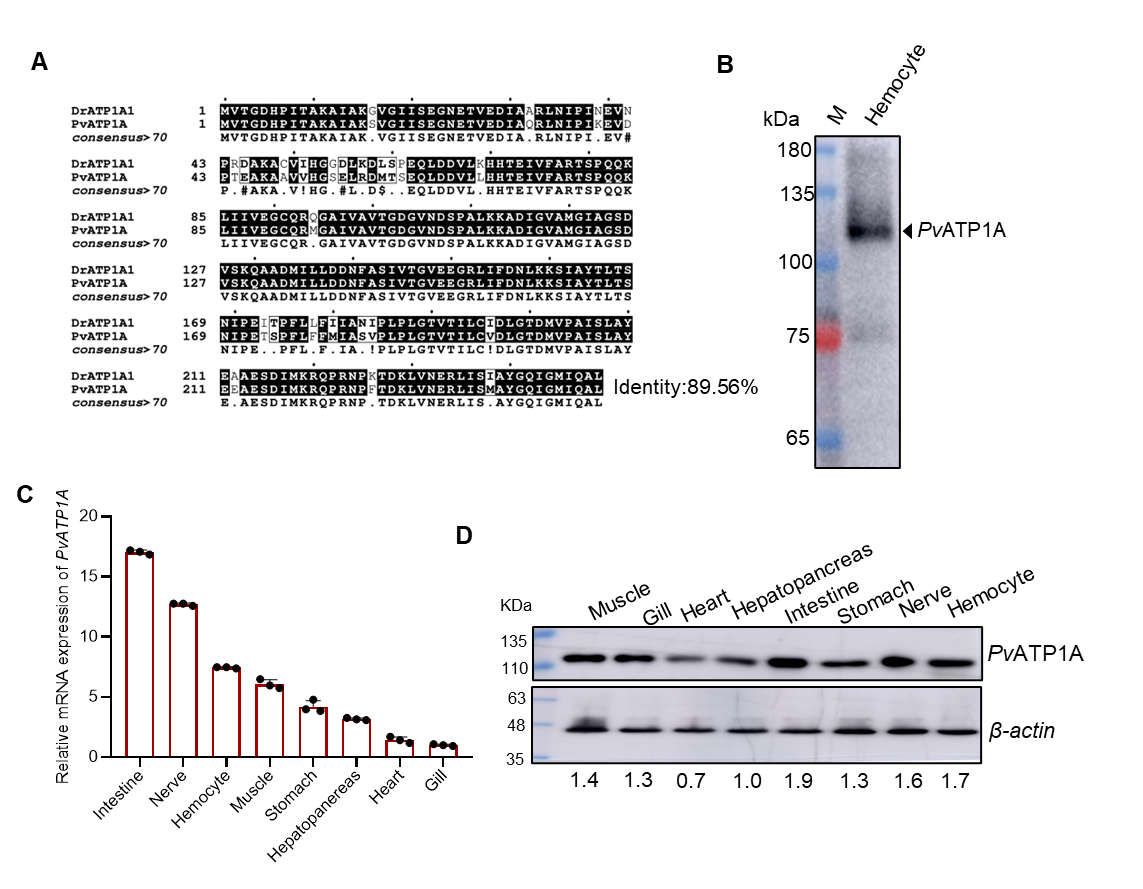
**

**Fig. S1 Tissue distribution analysis of *Pv*ATP1A. (A and B)** Antibody specificity analysis of *Danio rerio* ATP1A antibody (anti-*Dr*ATP1A) for detecting *Pv*ATP1A. Sequence identity between the anti-*Dr*ATP1A immunogen and the corresponding *Pv*ATP1A sequence was evaluated using DNAMAN software (A). Antibody specificity for *Pv*ATP1A detection in hemocytes was confirmed via Western blot analysis (B). M: Protein marker. **(C and D)** Expression profile of *Pv*ATP1A mRNA and protein across shrimp tissues. RNA and protein were extracted from hemocytes, gill, hepatopancreas, heart, nerve, muscle, stomach, and intestine, and analyzed using qPCR (C) and Western blot (D).

**
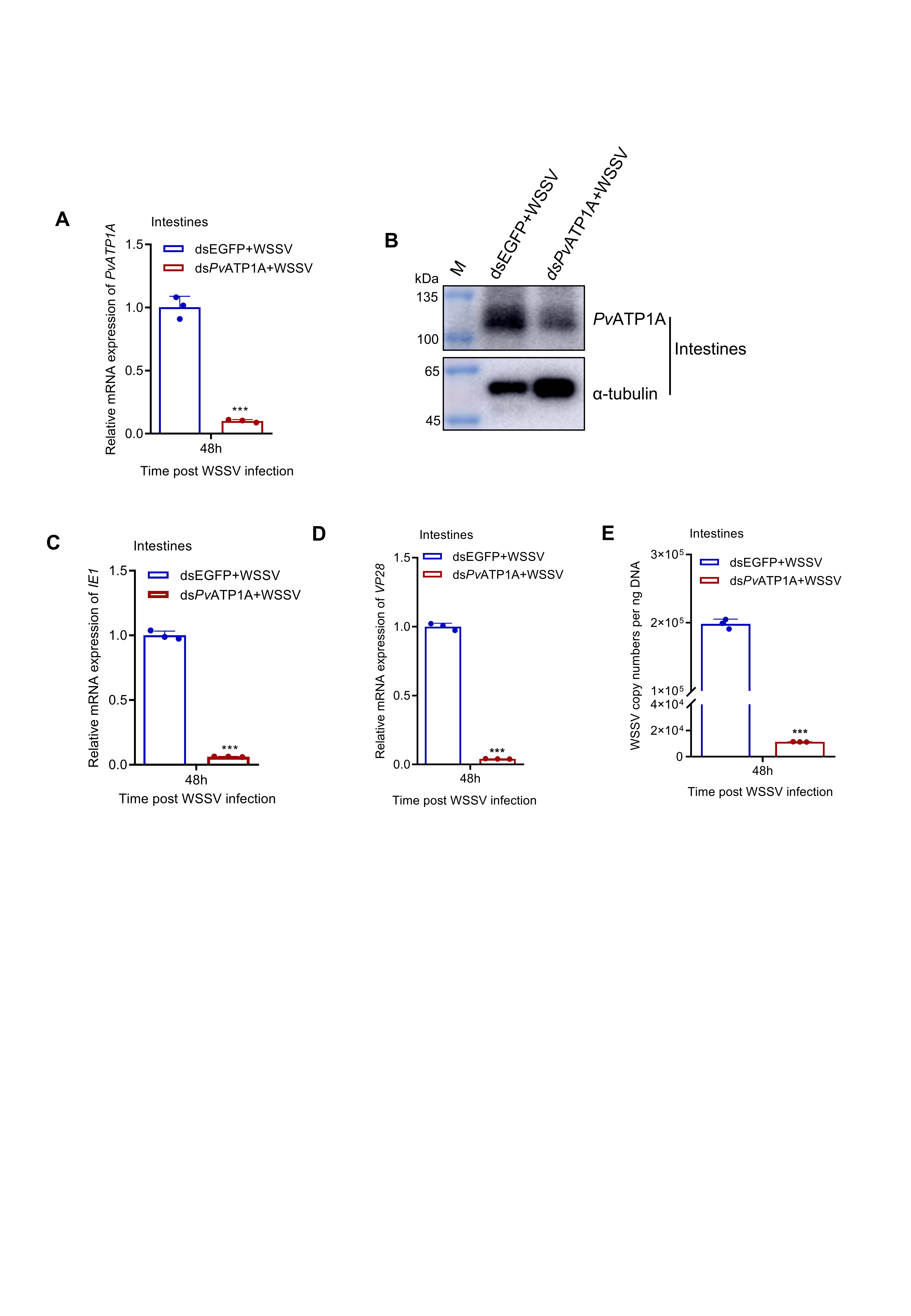
**

**Fig. S2 *Pv*ATP1A knockdown reduces WSSV infection in intestines.**  (**A and B**) Analysis of *Pv*ATP1A knockdown efficiency in intestines. Shrimps were injected with dsEGFP or ds*Pv*ATP1A, followed by WSSV infection 48 hours post-dsRNA injection. Intestines were harvested 48 hours post-infection to assess *Pv*ATP1A mRNA (A) and protein (B) levels using qPCR and Western blot, respectively. M: Protein marker.
(**C and D**) Quantification of WSSV gene expression in intestines. mRNA levels of WSSV genes *IE1* (C) and *VP28* (D) were measured via qPCR following *Pv*ATP1A knockdown. **(E**) WSSV copy number quantification post-*Pv*ATP1A knockdown by qPCR. Statistical significance was determined using a two-tailed Student’s t-test. ****P* < 0.001.


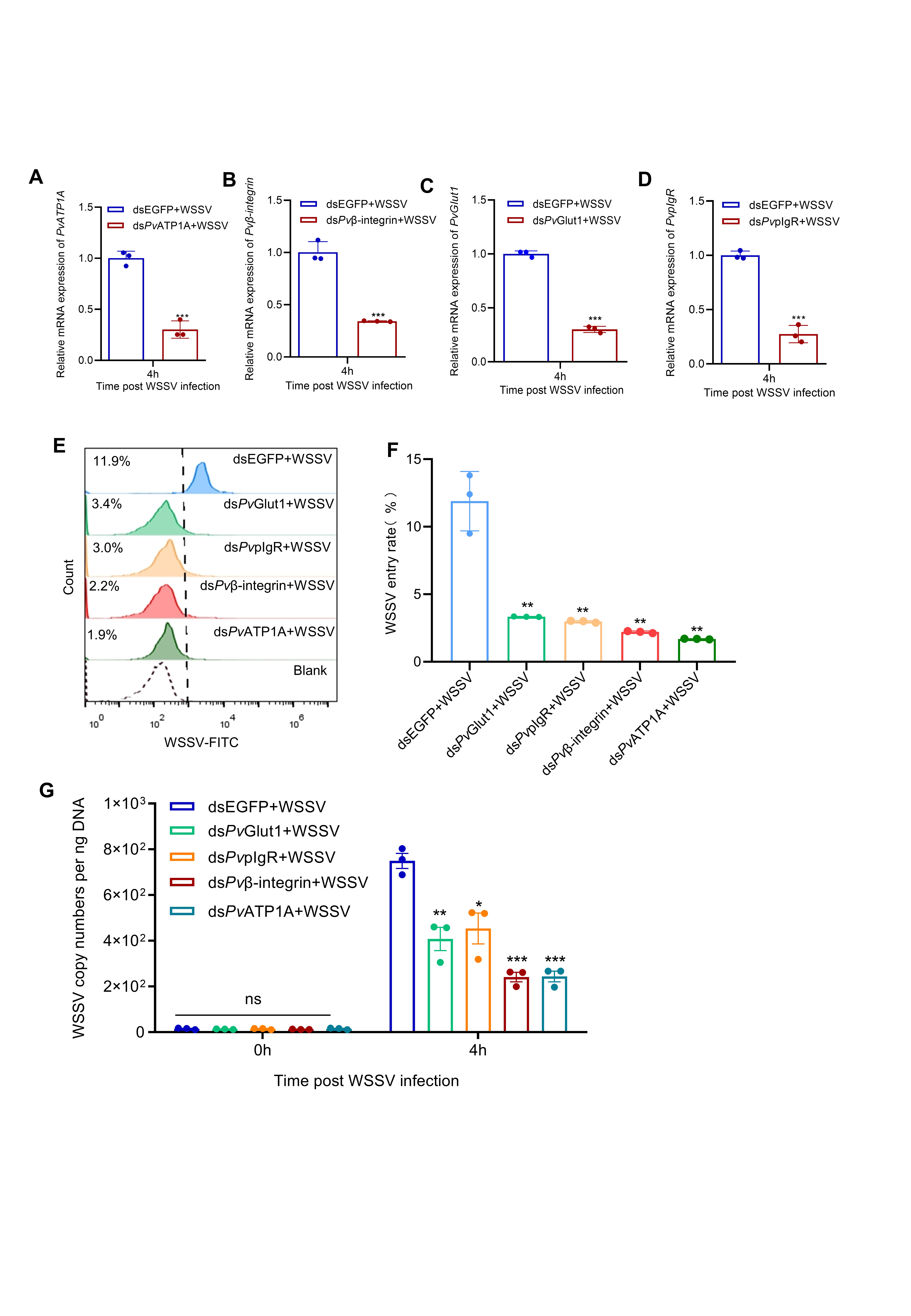


**Fig. S3 Comparative analysis of *Pv*ATP1A with other identified receptors in facilitating WSSV entry. (A-D**) Knockdown efficiency of *Pv*ATP1A and other receptors. Shrimps were injected with ds*Pv*ATP1A (A), ds*Pv*β-integrin (B), ds*Pv*Glut1 (C), or ds*Pv*pIgR (D), with dsEGFP as the control. WSSV particles were introduced 24 hours post-dsRNA injection, and hemocytes were harvested 4 hours post-infection for RNA extraction and qPCR analysis. (**E-G**) Assessment of WSSV entry following receptor knockdown. FITC-labeled or unlabeled WSSV particles were injected into shrimp pre- and post-receptor knockdown, and hemocytes were analyzed for WSSV entry using flow cytometry (E, F) and qPCR (G). Representative flow cytometry data are shown in (E), and the entry rate is quantified in (F) based on three independent replicates. Statistical analysis was conducted using a two-tailed Student’s t-test. * *P* < 0.05, ** *P* < 0.01, and *** *P* < 0.001.

**
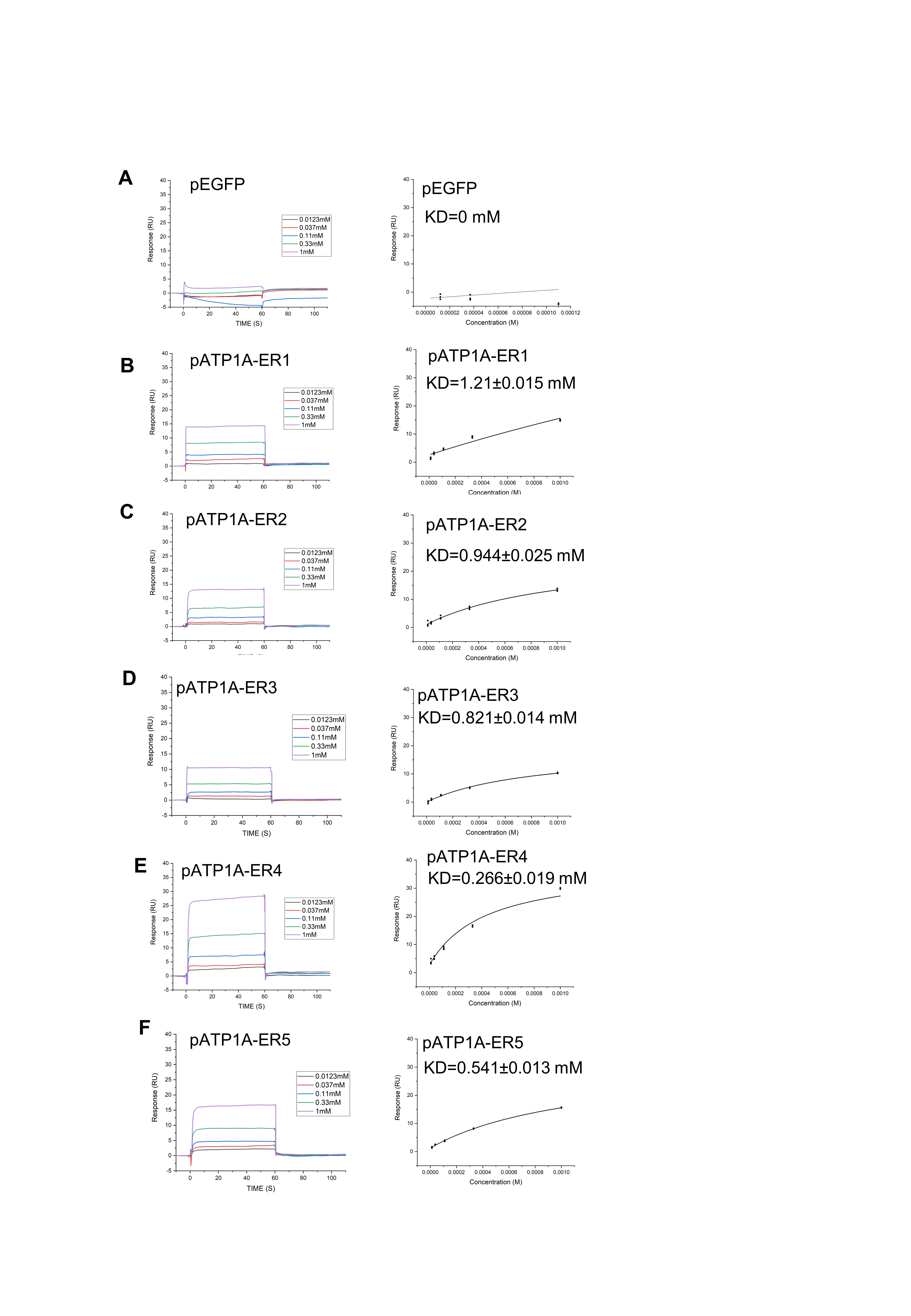
**

**Fig. S4 Surface plasmon resonance (SPR) analysis of interactions between *Pv*ATP1A extracellular peptides and VP28.** SPR experiments were performed on a Biacore T200 system using a CM5 sensor chip immobilized with recombinant VP28 protein. Synthetic peptides, including pEGFP (A), pATP1A-ER1 (B), pATP1A-ER2 (C), pATP1A-ER3 (D), pATP1A-ER4 (E), and pATP1A-ER5 (F), were injected over the chip. Assays were conducted in triplicate, with a representative sensorgram displaying interaction kinetics (right) and steady-state affinity (left).

**
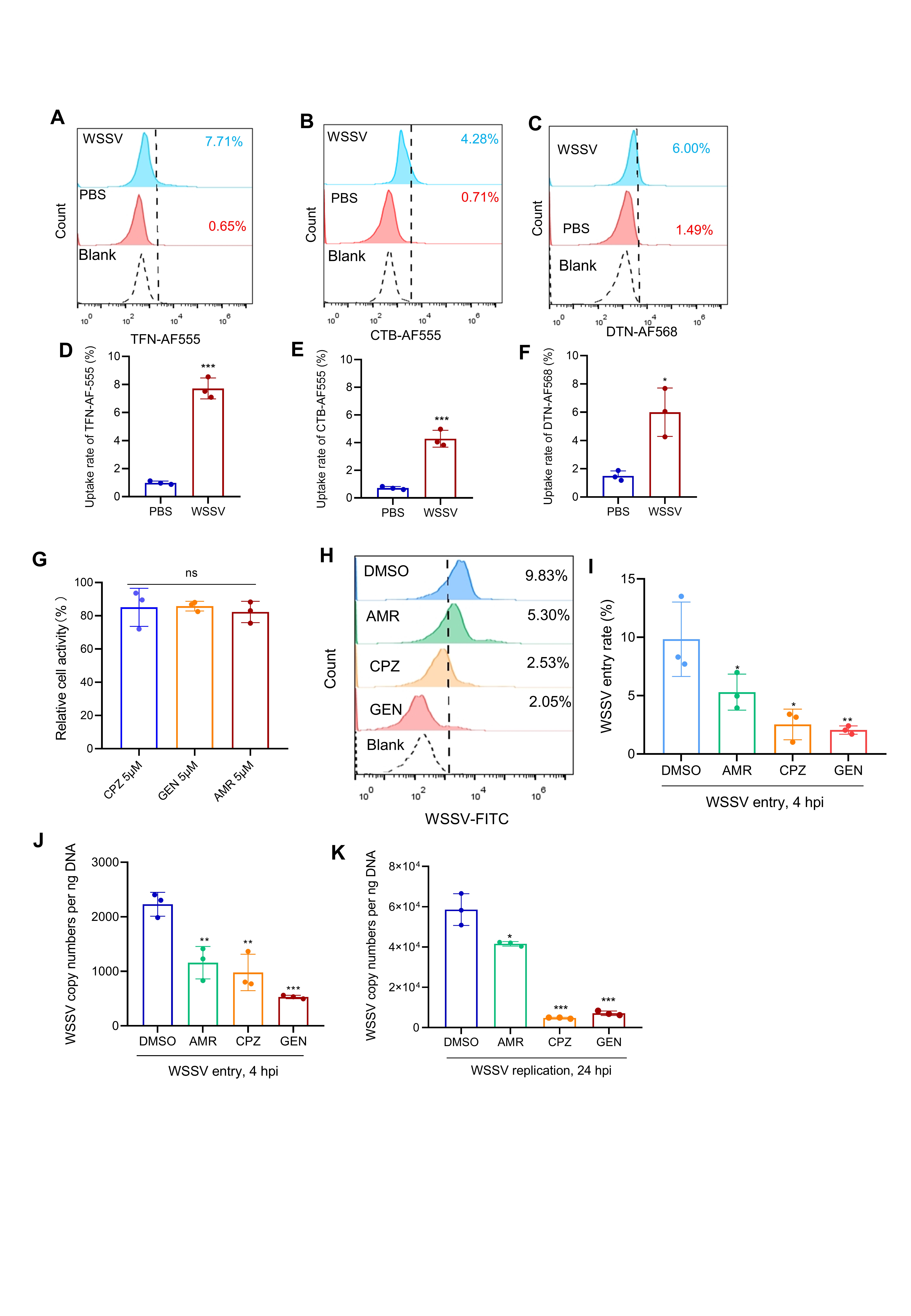
**

**Fig. S5 WSSV entry into shrimp hemocytes through multiple endocytic pathways.** (**A-F**) Identification of endocytic pathways triggered by WSSV. Shrimps were injected with either WSSV or PBS (negative control), and hemocytes were harvested 4 hours post-infection. Hemocytes were incubated with endocytic markers TFN-AF555, CTB-AF555, and DTN-AF568, and uptake rates were analyzed by flow cytometry. Panels (A-C) display representative flow cytometry images for the uptake of TFN-AF555, CTB-AF555, and DTN-AF568, respectively. Quantified uptake rates from three independent experiments are shown in panels (D-F). (**G-J**) Impact of endocytic inhibitors on WSSV entry. Shrimps were injected with the inhibitors CPZ, GEN, or AMR, and hemocytes were collected 2 hours later for cytotoxicity analysis using the CCK8 kit (G). FITC-labeled or unlabeled WSSV particles were subsequently injected, and hemocytes were harvested 4 hours post-infection for flow cytometry (H) and qPCR (J) to assess WSSV entry. Data from three independent flow cytometry assays are presented in panel (I). (**K**) Effect of endocytic inhibitors on WSSV replication. Shrimps were injected with WSSV particles following inhibitor treatment, and hemocytes were collected 24 hours post-infection for WSSV copy number quantification via qPCR. Statistical significance was determined using a two-tailed Student’s t-test. * *P* < 0.05, ** *P* < 0.01, and *** *P* < 0.001.

**Table S1 LC-MS/MS results from BioID-pull down samples**

**a) Proteins identified in GST-BirA* group**

| **Accession** | **Description** | **Peptides** | **Unique Peptides** | **PSMs** | **Coverage**  **[%]** | **Score Sequest HT** |
| --- | --- | --- | --- | --- | --- | --- |
| ROT67005.1 | hypothetical protein C7M84_014932 | 5 | 5 | 6 | 2 | 16.62 |
| ROT85516.1 | putative ryanodine receptor 44F isoform X4 | 4 | 4 | 10 | 3 | 24.47 |
| ROT62087.1 | putative RING finger protein nhl-1 | 4 | 4 | 6 | 7 | 13.93 |
| ROT82296.1 | hypothetical protein C7M84_024534 | 4 | 4 | 4 | 2 | 8.85 |
| ROT73965.1 | hypothetical protein C7M84_007564 | 4 | 3 | 6 | 8 | 13.05 |
| ROT66991.1 | hypothetical protein C7M84_014942 | 3 | 3 | 59 | 7 | 134.61 |
| ROT82106.1 | Spectrin beta chain | 3 | 3 | 17 | 2 | 39.2 |
| ROT81450.1 | putative kinesin-like protein KIF12 | 3 | 3 | 16 | 13 | 34.16 |
| ROT81584.1 | hypothetical protein C7M84_025257 | 3 | 3 | 16 | 2 | 34.86 |
| ROT83153.1 | hypothetical protein C7M84_023669 | 3 | 3 | 13 | 3 | 28.86 |
| ROT79375.1 | putative ATP-binding cassette sub-family A member 3 | 3 | 3 | 11 | 6 | 29.29 |
| ROT62129.1 | hypothetical protein C7M84_020041 | 3 | 3 | 10 | 2 | 24.5 |
| ROT80795.1 | putative serine/threonine-protein phosphatase 6 regulatory ankyrin repeat subunit B-like | 3 | 3 | 9 | 3 | 21.02 |
| ROT71177.1 | hypothetical protein C7M84_010509 | 3 | 3 | 8 | 7 | 17.66 |
| ROT80965.1 | Abhydrolase domain-containing protein 8 | 3 | 3 | 8 | 5 | 21.19 |
| ROT67836.1 | putative transmembrane protease serine 9-like | 3 | 3 | 7 | 11 | 15.95 |
| ROT74080.1 | hypothetical protein C7M84_007436 | 3 | 3 | 7 | 2 | 19.49 |
| ROT75576.1 | putative molybdenum cofactor sulfurase 1 isoform X2 | 3 | 3 | 7 | 5 | 18.35 |
| ROT75605.1 | hypothetical protein C7M84_005857 | 3 | 3 | 6 | 11 | 13.08 |
| ROT77097.1 | putative DNA polymerase theta-like isoform X1 | 3 | 3 | 5 | 4 | 14.56 |
| ROT68675.1 | hypothetical protein C7M84_013157 | 3 | 3 | 5 | 12 | 14.12 |
| ROT70240.1 | Filamin-A | 3 | 3 | 4 | 12 | 10.77 |
| ROT77602.1 | putative adenomatous polyposis coli protein-like isoform X3 | 3 | 3 | 4 | 2 | 10.37 |
| ROT66461.1 | hypothetical protein C7M84_015508 | 3 | 3 | 4 | 11 | 8.83 |
| ROT74598.1 | hypothetical protein C7M84_006933 [Penaeus vannamei] | 3 | 3 | 4 | 8 | 9.26 |
| ROT71671.1 | HEAT repeat-containing protein 1 | 3 | 3 | 3 | 3 | 7.79 |
| ROT78859.1 | hypothetical protein C7M84_002421 | 3 | 3 | 3 | 3 | 7.75 |
| ROT76308.1 | Membrane-associated guanylate kinase, WW and PDZ domain-containing protein 2 | 3 | 3 | 3 | 3 | 6.62 |
| ROT81616.1 | hypothetical protein C7M84_025230 | 3 | 3 | 3 | 2 | 7.28 |
| ROT64321.1 | putative stress protein DDR48-like | 3 | 3 | 3 | 6 | 7.23 |
| ROT85493.1 | Leucine-rich repeat serine/threonine-protein kinase 1 | 3 | 3 | 3 | 2 | 6.99 |
| ROT74448.1 | putative ankyrin-1-like | 3 | 3 | 3 | 13 | 6.92 |
| ROT73064.1 | neurofibromin | 3 | 3 | 3 | 3 | 7.31 |
| ROT76862.1 | putative tyrosine-protein kinase-like otk isoform X2 | 3 | 3 | 3 | 5 | 6.67 |
| ROT65124.1 | neurofilament protein | 3 | 3 | 3 | 3 | 6.75 |
| ROT68445.1 | Collagen-like protein 2 | 3 | 3 | 3 | 11 | 8.95 |
| ROT66242.1 | putative rootletin | 3 | 3 | 3 | 2 | 6.69 |
| ROT63389.1 | hemocyanin subunit L2 | 3 | 2 | 7 | 4 | 26.08 |
| ROT61916.1 | hypothetical protein C7M84_020263 | 2 | 2 | 60 | 9 | 137.23 |
| ROT64238.1 | Mitochondrial ribonuclease P protein 1-like protein | 2 | 2 | 31 | 10 | 84.49 |
| ROT73582.1 | putative janus kinase and microtubule-interacting protein 3 | 2 | 2 | 30 | 3 | 66.57 |
| ROT62328.1 | heat shock protein 21 | 2 | 2 | 21 | 3 | 44.14 |
| ROT63507.1 | hypothetical protein C7M84_018607 | 2 | 2 | 20 | 7 | 50.8 |
| ROT65617.1 | glucose-6-phosphate isomerase | 2 | 2 | 20 | 4 | 42.54 |
| ROT64876.1 | Hexosaminidase domain-containing protein | 2 | 2 | 20 | 9 | 51.41 |
| ROT74594.1 | putative neuroblast differentiation-associated protein AHNAK-like | 2 | 2 | 16 | 3 | 37.25 |
| ROT72037.1 | putative synaptonemal complex protein 1-like | 2 | 2 | 15 | 2 | 43.52 |
| ROT79688.1 | Tyrosine-protein phosphatase non-receptor type 23 | 2 | 2 | 14 | 2 | 35.98 |
| ROT77868.1 | putative spatacsin isoform X1 | 2 | 2 | 13 | 2 | 29.07 |
| ROT66095.1 | AP2-associated protein kinase 1 | 2 | 2 | 12 | 4 | 25.4 |
| ROT74150.1 | hypothetical protein C7M84_007364 | 2 | 2 | 10 | 12 | 24.24 |
| ROT69951.1 | putative FH1/FH2 domain-containing protein 3 | 2 | 2 | 10 | 2 | 21.36 |
| ROT77912.1 | hypothetical protein C7M84_003392 | 2 | 2 | 10 | 9 | 24.09 |
| ROT63726.1 | Zinc finger FYVE domain-containing protein 9 | 2 | 2 | 9 | 8 | 24.96 |
| ROT79955.1 | hypothetical protein C7M84_001325 | 2 | 2 | 9 | 8 | 21.02 |
| ROT73083.1 | Ral GTPase-activating protein subunit alpha-2 | 2 | 2 | 9 | 3 | 24.77 |
| ROT81045.1 | hypothetical protein C7M84_000204 | 2 | 2 | 9 | 2 | 23.3 |
| ROT77625.1 | Golgi-specific brefeldin A-resistance guanine nucleotide exchange factor 1 | 2 | 2 | 8 | 2 | 19.25 |
| ROT77065.1 | zinc finger protein | 2 | 2 | 8 | 4 | 21.98 |
| ROT76848.1 | hypothetical protein C7M84_004550 | 2 | 2 | 8 | 2 | 19.36 |
| ROT83440.1 | putative calpain-A-like isoform X5 | 2 | 2 | 7 | 12 | 18.87 |
| ROT73906.1 | glycosyltransferase PglE | 2 | 2 | 7 | 6 | 17.85 |
| ROT63707.1 | Alpha-(1,6)-fucosyltransferase | 2 | 2 | 7 | 14 | 19.74 |
| ROT76961.1 | putative muscle M-line assembly protein unc-89-like isoform | 2 | 2 | 7 | 3 | 15.87 |
| ROT63741.1 | putative proline-rich protein 21 | 2 | 2 | 7 | 13 | 15.42 |
| ROT76793.1 | PHD finger protein rhinocero | 2 | 2 | 6 | 1 | 12.52 |
| ROT76287.1 | hypothetical protein C7M84_005128 | 2 | 2 | 6 | 10 | 13.37 |
| ROT77599.1 | hypothetical protein C7M84_003740 | 2 | 2 | 6 | 2 | 14.12 |
| ROT75125.1 | putative myosin light chain kinase | 2 | 2 | 6 | 1 | 12.54 |
| ROT72893.1 | putative transformation/transcription domain-associated protein | 2 | 2 | 6 | 1 | 11.68 |
| ROT61904.1 | hypothetical protein C7M84_020277 | 2 | 2 | 6 | 5 | 13.02 |
| ROT80323.1 | hypothetical protein C7M84_000940 | 2 | 2 | 6 | 4 | 15.38 |
| ROT63913.1 | putative midnolin-like | 2 | 2 | 6 | 7 | 14.39 |
| ROT71964.1 | hypothetical protein C7M84_009674 | 2 | 2 | 6 | 2 | 13.13 |
| ROT83262.1 | Protein Hook-like protein | 2 | 2 | 5 | 7 | 12.11 |
| ROT63411.1 | hypothetical protein C7M84_018721 | 2 | 2 | 5 | 3 | 10.45 |
| ROT71391.1 | Cell division cycle 7-related protein kinase | 2 | 2 | 5 | 3 | 14.94 |
| ROT73090.1 | hypothetical protein C7M84_008487 | 2 | 2 | 5 | 2 | 10.38 |
| ROT83695.1 | putative dystrophin, isoforms A/C/F/G/H isoform X7 | 2 | 2 | 5 | 1 | 10.42 |
| ROT60986.1 | hypothetical protein C7M84_021295 | 2 | 2 | 5 | 4 | 13.17 |
| ROT82680.1 | WD repeat-containing protein 48 | 2 | 2 | 5 | 10 | 14.22 |
| ROT69515.1 | hypothetical protein C7M84_012282 | 2 | 2 | 5 | 4 | 11.24 |
| ROT74447.1 | hypothetical protein C7M84_007050 | 2 | 2 | 5 | 6 | 11.54 |
| ROT69205.1 | hypothetical protein C7M84_012677 | 2 | 2 | 5 | 2 | 10.58 |
| ROT62735.1 | hypothetical protein C7M84_019403 | 2 | 2 | 5 | 3 | 12.15 |
| ROT83614.1 | putative WD repeat-containing protein 19-like | 2 | 2 | 5 | 2 | 11.04 |
| ROT65042.1 | hypothetical protein C7M84_017010 | 2 | 2 | 5 | 2 | 13.45 |
| ROT82054.1 | hypothetical protein C7M84_024781 | 2 | 2 | 5 | 20 | 12.17 |
| ROT69632.1 | putative receptor-type tyrosine-protein phosphatase S-like | 2 | 2 | 5 | 4 | 12.62 |
| ROT69861.1 | hypothetical protein C7M84_011907 | 2 | 2 | 5 | 3 | 10.81 |
| ROT66270.1 | hypothetical protein C7M84_015732 | 2 | 2 | 5 | 2 | 12.97 |
| ROT81820.1 | putative THUMP domain-containing protein 3 | 2 | 2 | 5 | 5 | 10.82 |
| ROT73809.1 | hypothetical protein C7M84_007719 | 2 | 2 | 5 | 4 | 12.37 |
| ROT80318.1 | putative chitinase 10 isoform X1 | 2 | 2 | 4 | 2 | 10.08 |
| ROT81833.1 | Structural maintenance of chromosomes protein 1A | 2 | 2 | 4 | 3 | 10.24 |
| ROT74196.1 | hypothetical protein C7M84_007313 | 2 | 2 | 4 | 1 | 8.79 |
| ROT83507.1 | hypothetical protein C7M84_023303 | 2 | 2 | 4 | 9 | 10.75 |
| ROT83108.1 | Glutamate synthase | 2 | 2 | 4 | 2 | 9.2 |
| ROT77906.1 | putative eukaryotic translation initiation factor 2-alpha kinase | 2 | 2 | 4 | 3 | 8.86 |
| ROT61076.1 | putative mucin-5AC | 2 | 2 | 4 | 2 | 8.41 |
| ROT78160.1 | hypothetical protein C7M84_003123 | 2 | 2 | 4 | 10 | 10.17 |
| ROT75636.1 | Glycoprotein-N-acetylgalactosamine 3-beta-galactosyltransferase 1 | 2 | 2 | 4 | 9 | 9.3 |
| ROT69333.1 | Glutamate receptor delta-1 subunit | 2 | 2 | 4 | 8 | 8.79 |
| ROT80891.1 | hypothetical protein C7M84_000350 | 2 | 2 | 4 | 0 | 8.26 |
| ROT65240.1 | Translational activator GCN1 | 2 | 2 | 4 | 2 | 9.4 |
| ROT74992.1 | adenylate cyclase | 2 | 2 | 4 | 15 | 10.03 |
| ROT77895.1 | hypothetical protein C7M84_003410 | 2 | 2 | 4 | 8 | 8.79 |
| ROT85135.1 | hypothetical protein C7M84_021163 | 2 | 2 | 4 | 9 | 10.31 |
| ROT64049.1 | hypothetical protein C7M84_018028 | 2 | 2 | 4 | 3 | 8.19 |
| ROT63911.1 | trehalose transporter 1-2 | 2 | 2 | 4 | 7 | 8.85 |
| ROT66243.1 | hypothetical protein C7M84_015765 | 2 | 2 | 4 | 7 | 10.72 |
| ROT73388.1 | hypothetical protein C7M84_008160 | 2 | 2 | 4 | 5 | 12.32 |
| ROT62064.1 | hypothetical protein C7M84_020102 | 2 | 2 | 4 | 5 | 9.5 |
| ROT83285.1 | putative neural-cadherin | 2 | 2 | 4 | 2 | 8.52 |
| ROT75811.1 | Protein ELYS | 2 | 2 | 4 | 1 | 9.97 |
| ROT86046.1 | hypothetical protein C7M84_015234 | 2 | 2 | 3 | 5 | 7.23 |
| ROT78043.1 | hypothetical protein C7M84_003266 | 2 | 2 | 3 | 2 | 7.97 |
| ROT64889.1 | Histone-lysine N-methyltransferase MLL2 | 2 | 2 | 3 | 4 | 6.32 |
| ROT62639.1 | hypothetical protein C7M84_019503 | 2 | 2 | 3 | 10 | 7.36 |
| ROT81394.1 | putative transforming acidic coiled-coil-containing protein 1 | 2 | 2 | 3 | 2 | 6.79 |
| ROT63347.1 | hypothetical protein C7M84_018752 | 2 | 2 | 3 | 6 | 6.51 |
| ROT81932.1 | hypothetical protein C7M84_024919 | 2 | 2 | 3 | 10 | 6.93 |
| ROT79695.1 | hypothetical protein C7M84_001583 | 2 | 2 | 3 | 6 | 8.45 |
| ROT62742.1 | WD40 repeat-containing protein SMU1 | 2 | 2 | 3 | 11 | 7.73 |
| ROT66502.1 | Sodium channel protein 60E | 2 | 2 | 3 | 3 | 7.52 |
| ROT64061.1 | putative serine/threonine-protein kinase SMG1 | 2 | 2 | 3 | 3 | 6.82 |
| ROT61808.1 | hypothetical protein C7M84_020383 | 2 | 2 | 3 | 5 | 6.71 |
| ROT70093.1 | putative glutamate-gated chloride channel-like | 2 | 2 | 3 | 4 | 7.17 |
| ROT72475.1 | hypothetical protein C7M84_009145 | 2 | 2 | 3 | 1 | 7.14 |
| ROT80443.1 | DnaJ-like protein subfamily B member 9 | 2 | 2 | 3 | 6 | 7.49 |
| ROT73609.1 | cell surface protein | 2 | 2 | 3 | 3 | 8.06 |
| ROT61896.1 | hypothetical protein C7M84_020288 | 2 | 2 | 3 | 9 | 8.5 |
| ROT68484.1 | hypothetical protein C7M84_013375 | 2 | 2 | 3 | 7 | 7.26 |
| ROT70758.1 | Dystroglycan | 2 | 2 | 3 | 4 | 7.58 |
| ROT77071.1 | hypothetical protein C7M84_004303 | 2 | 2 | 3 | 6 | 6.93 |
| ROT65329.1 | hypothetical protein C7M84_016705 | 2 | 2 | 3 | 8 | 6.68 |
| ROT67568.1 | putative protein ECT2 isoform X6 | 2 | 2 | 3 | 7 | 7.92 |
| ROT71435.1 | hypothetical protein C7M84_010253 | 2 | 2 | 3 | 7 | 7.56 |
| ROT79403.1 | Cytochrome P450 3A13 | 2 | 2 | 3 | 9 | 7 |
| ROT78099.1 | MKKK7-interacting protein 1 | 2 | 2 | 3 | 12 | 8.74 |
| ROT77847.1 | Sorbin and SH3 domain-containing protein | 2 | 2 | 3 | 6 | 6.2 |
| ROT85378.1 | DNA-binding protein RFX7 | 2 | 2 | 3 | 7 | 8.79 |
| ROT76749.1 | hypothetical protein C7M84_004663 | 2 | 2 | 3 | 7 | 6.4 |
| ROT85099.1 | putative splicing factor 3B subunit 2 isoform X3 | 2 | 2 | 3 | 7 | 6.88 |
| ROT76154.1 | heat shock protein 40 | 2 | 2 | 3 | 8 | 9.39 |
| ROT63313.1 | putative transmembrane channel-like protein 3 | 2 | 2 | 3 | 2 | 6.84 |
| ROT71770.1 | hypothetical protein C7M84_009887 | 2 | 2 | 3 | 8 | 6.39 |
| ROT69726.1 | hypothetical protein C7M84_012048 | 2 | 2 | 3 | 2 | 6.42 |
| ROT73285.1 | hypothetical protein C7M84_008284 | 2 | 2 | 3 | 10 | 7.8 |
| ROT68692.1 | putative collagen alpha-1(VIII) chain-like isoform X4 | 2 | 2 | 3 | 3 | 8.3 |
| ROT62178.1 | hypothetical protein C7M84_019993 | 2 | 2 | 3 | 1 | 6.15 |
| ROT84842.1 | putative DNA helicase Ino80 | 2 | 2 | 3 | 4 | 8.03 |
| ROT70986.1 | hypothetical protein C7M84_010710 | 2 | 2 | 3 | 6 | 8.05 |
| ROT72823.1 | Kinesin-like protein costa | 2 | 2 | 3 | 3 | 8.11 |
| ROT61314.1 | hypothetical protein C7M84_020919 | 2 | 2 | 3 | 6 | 6.13 |
| ROT74727.1 | hypothetical protein C7M84_006752 | 2 | 2 | 3 | 6 | 7.85 |
| ROT85175.1 | hypothetical protein C7M84_020692 | 2 | 2 | 3 | 4 | 7.23 |
| ROT64047.1 | putative NACHT, LRR and PYD domains-containing protein | 2 | 2 | 3 | 3 | 6.48 |
| ROT61796.1 | hypothetical protein C7M84_020393 | 2 | 2 | 3 | 8 | 8.28 |
| ROT70397.1 | hypothetical protein C7M84_011323 | 2 | 2 | 3 | 4 | 6.79 |
| ROT62011.1 | heat shock protein 70 | 2 | 2 | 3 | 8 | 7.02 |
| ROT74479.1 | hypothetical protein C7M84_007020 | 2 | 2 | 3 | 3 | 7.32 |
| ROT67791.1 | putative histone-lysine N-methyltransferase | 2 | 2 | 3 | 2 | 6.74 |
| ROT63504.1 | hypothetical protein C7M84_018604 | 2 | 2 | 3 | 5 | 6.29 |
| ROT66603.1 | hypothetical protein C7M84_015402 | 2 | 2 | 2 | 2 | 4.74 |
| ROT62519.1 | putative olfactory receptor 11A1-like | 2 | 2 | 2 | 5 | 4.28 |
| ROT77852.1 | Ficolin-1 | 2 | 2 | 2 | 6 | 4.62 |
| ROT63807.1 | hypothetical protein C7M84_018283 | 2 | 2 | 2 | 3 | 4.74 |
| ROT79351.1 | kinesin heavy chain | 2 | 2 | 2 | 3 | 4.08 |
| ROT63882.1 | hypothetical protein C7M84_018199 | 2 | 2 | 2 | 3 | 4.82 |
| ROT83496.1 | Phosphoinositide 3-kinase regulatory subunit 4 | 2 | 2 | 2 | 4 | 5.81 |
| ROT75499.1 | hypothetical protein C7M84_005956 | 2 | 2 | 2 | 11 | 6.25 |
| ROT76564.1 | hypothetical protein C7M84_004848 | 2 | 2 | 2 | 29 | 4.88 |
| ROT62401.1 | Chaperone protein | 2 | 2 | 2 | 12 | 5.62 |
| ROT76885.1 | hypothetical protein C7M84_004502 | 2 | 2 | 2 | 8 | 4.84 |
| ROT67741.1 | hypothetical protein C7M84_014164 | 2 | 2 | 2 | 3 | 4.89 |
| ROT61959.1 | hypothetical protein C7M84_020208 | 2 | 2 | 2 | 2 | 4.29 |
| ROT76746.1 | hypothetical protein C7M84_004660 | 2 | 2 | 2 | 2 | 4.5 |
| ROT80442.1 | putative vacuolar protein sorting-associated protein 13B-like | 2 | 2 | 2 | 1 | 4.6 |
| ROT62868.1 | Calcium-binding mitochondrial carrier protein SCaMC | 2 | 2 | 2 | 12 | 4.77 |
| ROT61213.1 | putative Ecdysone receptor | 2 | 2 | 2 | 4 | 4.74 |
| ROT62974.1 | hypothetical protein C7M84_019157 | 2 | 2 | 2 | 3 | 4.73 |
| ROT78078.1 | hypothetical protein C7M84_003216 | 2 | 2 | 2 | 4 | 4.67 |
| ROT79745.1 | hypothetical protein C7M84_001541 | 2 | 2 | 2 | 2 | 4.71 |
| ROT80511.1 | hypothetical protein C7M84_000747 | 2 | 2 | 2 | 7 | 4.08 |
| ROT75976.1 | hypothetical protein C7M84_005462 | 2 | 2 | 2 | 9 | 5.33 |
| ROT63255.1 | Spectrin alpha chain | 2 | 2 | 2 | 1 | 4.72 |
| ROT83725.1 | putative zinc finger protein ZPR1 isoform X1 | 2 | 2 | 2 | 10 | 4.55 |
| ROT80144.1 | hypothetical protein C7M84_001143 | 2 | 2 | 2 | 1 | 5.28 |
| ROT64498.1 | putative Down syndrome cell adhesion molecule-like protein | 2 | 2 | 2 | 9 | 5.27 |
| ROT78633.1 | hypothetical protein C7M84_002645 | 2 | 2 | 2 | 8 | 4.56 |
| ROT65479.1 | Glycine dehydrogenase | 2 | 2 | 2 | 2 | 4.16 |
| ROT78654.1 | notch protein | 2 | 2 | 2 | 3 | 5.47 |
| ROT74637.1 | putative cleavage and polyadenylation specificity factor | 2 | 2 | 2 | 4 | 4.66 |
| ROT69760.1 | hypothetical protein C7M84_012007 | 2 | 2 | 2 | 4 | 4.84 |
| ROT63339.1 | hypothetical protein C7M84_018790 | 2 | 2 | 2 | 1 | 4.04 |
| ROT76182.1 | putative dentin sialophosphoprotein | 2 | 2 | 2 | 5 | 5.44 |
| ROT71899.1 | putative dynein heavy chain 10, axonemal | 2 | 2 | 2 | 4 | 4.58 |
| ROT75096.1 | putative beta,beta-carotene 9 | 2 | 2 | 2 | 14 | 5.32 |
| ROT72221.1 | double-stranded RNA-specific adenosine deaminase | 2 | 2 | 2 | 19 | 5.85 |
| ROT62117.1 | putative targeting protein for Xklp2-like isoform X1 | 2 | 2 | 2 | 3 | 4.31 |
| ROT66655.1 | hypothetical protein C7M84_015309 | 2 | 2 | 2 | 5 | 4.85 |
| ROT67593.1 | putative coiled-coil domain-containing protein 27 | 2 | 2 | 2 | 1 | 4.16 |
| ROT65959.1 | hypothetical protein C7M84_016051 | 2 | 2 | 2 | 24 | 5.08 |
| ROT84120.1 | hypothetical protein C7M84_022696 | 2 | 2 | 2 | 5 | 5.25 |
| ROT85531.1 | hypothetical protein C7M84_012487 | 2 | 2 | 2 | 4 | 5.51 |
| ROT62699.1 | hypothetical protein C7M84_019414 | 2 | 2 | 2 | 12 | 6.64 |
| ROT68318.1 | Transient receptor potential channel pyrexia | 2 | 2 | 2 | 2 | 5.3 |
| ROT80410.1 | Beta-mannosidase | 2 | 2 | 2 | 5 | 4.79 |
| ROT77110.1 | putative aminopeptidase N | 2 | 2 | 2 | 6 | 4.28 |
| ROT62695.1 | hypothetical protein C7M84_019444 | 2 | 2 | 2 | 1 | 4.6 |
| ROT84889.1 | prophenoloxidase activating enzyme 2 | 2 | 2 | 2 | 14 | 5.7 |
| ROT64692.1 | hypothetical protein C7M84_017355 | 2 | 2 | 2 | 9 | 5.32 |
| ROT81420.1 | medium-chain specific acyl-CoA dehydrogenase | 2 | 2 | 2 | 13 | 5.4 |
| ROT80293.1 | hypothetical protein C7M84_000989 | 2 | 2 | 2 | 3 | 4.36 |
| ROT65473.1 | hypothetical protein C7M84_016552 | 2 | 2 | 2 | 1 | 6.03 |
| ROT64974.1 | hypothetical protein C7M84_017081 | 2 | 2 | 2 | 8 | 4.43 |
| ROT63725.1 | hypothetical protein C7M84_018378 | 2 | 2 | 2 | 7 | 6.27 |
| ROT82900.1 | Galactose-1-phosphate uridylyltransferase | 2 | 2 | 2 | 10 | 4.75 |
| ROT65318.1 | hypothetical protein C7M84_016717 | 2 | 2 | 2 | 2 | 4.32 |
| ROT69600.1 | hypothetical protein C7M84_012188 | 2 | 2 | 2 | 8 | 5.42 |
| ROT85129.1 | myosin Va | 2 | 2 | 2 | 4 | 5.13 |
| ROT69204.1 | alpha-I tubulin | 2 | 2 | 2 | 8 | 5.09 |
| ROT65200.1 | hypothetical protein C7M84_016851 | 2 | 2 | 2 | 5 | 5.5 |
| ROT68568.1 | 6-phosphofructo-2-kinase/fructose-2,6-bisphosphatase | 2 | 2 | 2 | 13 | 5.21 |
| ROT67182.1 | putative myotubularin-related protein 13 | 2 | 2 | 2 | 3 | 4.64 |
| ROT81913.1 | myosin heavy chain type a | 2 | 2 | 2 | 7 | 5.3 |
| ROT66061.1 | hypothetical protein C7M84_015957 | 2 | 2 | 2 | 2 | 4.32 |
| ROT65345.1 | hypothetical protein C7M84_016678 | 2 | 2 | 2 | 1 | 4.65 |
| ROT68415.1 | hypothetical protein C7M84_013429 | 2 | 2 | 2 | 2 | 4.63 |
| ROT69440.1 | putative fucosyltransferase | 2 | 2 | 2 | 6 | 4.1 |
| ROT62812.1 | hypothetical protein C7M84_019329 | 2 | 2 | 2 | 9 | 4.77 |
| ROT63478.1 | putative complex I intermediate-associated protein 30 | 2 | 2 | 2 | 15 | 5.79 |
| ROT81645.1 | putative fat-like cadherin-related tumor suppressor-like | 2 | 2 | 2 | 1 | 4.87 |
| ROT86026.1 | hypothetical protein C7M84_020413 | 2 | 2 | 2 | 7 | 4.69 |
| ROT82905.1 | hypothetical protein C7M84_023890 | 2 | 2 | 2 | 4 | 4.42 |
| ROT62266.1 | hypothetical protein C7M84_019906 | 2 | 2 | 2 | 5 | 4.03 |
| ROT73277.1 | putative ras-specific guanine nucleotide-releasing factor | 2 | 2 | 2 | 4 | 4.7 |
| ROT64723.1 | hypothetical protein C7M84_017328 | 2 | 2 | 2 | 6 | 5.67 |
| ROT67464.1 | hypothetical protein C7M84_014461 | 2 | 2 | 2 | 4 | 4.31 |
| ROT81685.1 | Zinc finger CCHC domain-containing protein 9 | 2 | 2 | 2 | 5 | 5.01 |
| ROT72346.1 | hypothetical protein C7M84_009256 | 2 | 2 | 2 | 4 | 4.74 |
| ROT77034.1 | hypothetical protein C7M84_004340 | 2 | 2 | 2 | 6 | 5.16 |
| ROT71755.1 | projectin | 2 | 2 | 2 | 3 | 5.41 |
| ROT61902.1 | hypothetical protein C7M84_020280 | 2 | 2 | 2 | 7 | 4.33 |
| ROT71318.1 | hypothetical protein C7M84_010368 | 2 | 2 | 2 | 8 | 5.46 |
| ROT79073.1 | Serine/threonine-protein kinase PLK4 | 2 | 2 | 2 | 5 | 5.79 |
| ROT73529.1 | putative protein abrupt isoform X2 | 2 | 2 | 2 | 13 | 4.88 |
| ROT68658.1 | hypothetical protein C7M84_013206 | 2 | 2 | 2 | 1 | 4.08 |
| ROT66085.1 | putative AP-3 complex subunit beta-1 | 2 | 2 | 2 | 2 | 4.33 |
| ROT71455.1 | hypothetical protein C7M84_010231 | 2 | 2 | 2 | 6 | 4.37 |
| ROT76271.1 | hypothetical protein C7M84_005165 | 2 | 2 | 2 | 2 | 4.25 |
| ROT75223.1 | Propionyl-CoA carboxylase alpha chain | 2 | 2 | 2 | 5 | 5.93 |
| ROT82996.1 | Cullin-2 | 2 | 2 | 2 | 6 | 4.76 |
| ROT62781.1 | Pre-mRNA-processing-splicing factor 8 | 2 | 2 | 2 | 2 | 4.62 |
| ROT81857.1 | putative serine/threonine-protein phosphatase 6 regulatory ankyrin repeat subunit A-like | 2 | 2 | 2 | 7 | 4.74 |
| ROT76510.1 | putative protein outspread-like | 2 | 2 | 2 | 3 | 5.13 |
| ROT62385.1 | ATP-binding cassette sub-family D member 2 | 2 | 2 | 2 | 3 | 4.8 |
| ROT69582.1 | hypothetical protein C7M84_012203 | 2 | 2 | 2 | 11 | 5.29 |
| ROT63695.1 | Protein farnesyltransferase subunit beta | 2 | 2 | 2 | 22 | 6.07 |
| ROT81470.1 | hypothetical protein C7M84_025371 | 2 | 2 | 2 | 7 | 4.65 |
| ROT61549.1 | hypothetical protein C7M84_020652 | 2 | 2 | 2 | 6 | 4.84 |
| ROT76305.1 | WNT2 protein | 2 | 2 | 2 | 3 | 4.06 |
| ROT78316.1 | hypothetical protein C7M84_002947 | 2 | 2 | 2 | 6 | 5.33 |
| ROT69596.1 | enzymatic polyprotein | 2 | 2 | 2 | 4 | 5.15 |
| ROT77750.1 | Down syndrome cell adhesion molecule isoform | 2 | 2 | 2 | 4 | 4.63 |
| ROT65174.1 | hypothetical protein C7M84_016876 | 2 | 2 | 2 | 1 | 5.46 |
| ROT85854.1 | hypothetical protein C7M84_005199 | 2 | 2 | 2 | 2 | 5.41 |
| ROT64935.1 | Huntingtin | 2 | 2 | 2 | 3 | 5.1 |

**b) Proteins identified in GST-BirA*-VP28 group**

| **Accession** | **Description** | **Peptides** | **Unique Peptides** | **PSMs** | **Coverage [%]** | **Score Sequest HT** |
| --- | --- | --- | --- | --- | --- | --- |
| ROT70788.1 | beta-actin | 8 | 5 | 31 | 31 | 89.94 |
| ROT83547.1 | hemocyanin | 6 | 5 | 18 | 15 | 54.29 |
| ROT65802.1 | hemocyanin | 5 | 2 | 15 | 11 | 64.56 |
| ROT75223.1 | Propionyl-CoA carboxylase alpha chain | 5 | 5 | 9 | 9 | 25.13 |
| ROT63389.1 | hemocyanin subunit L2 | 4 | 1 | 15 | 5 | 64.54 |
| ROT74594.1 | putative neuroblast differentiation-associated protein AHNAK-like | 4 | 4 | 15 | 5 | 34.91 |
| ROT74080.1 | hypothetical protein C7M84_007436 | 4 | 4 | 14 | 3 | 44.63 |
| ROT66128.1 | actin T2 | 4 | 2 | 13 | 18 | 30.45 |
| ROT69209.1 | actin 1 | 4 | 1 | 10 | 21 | 35.56 |
| ROT79823.1 | putative calcineurin-binding protein cabin-1 isoform X8 | 4 | 4 | 9 | 3 | 22.89 |
| ROT85516.1 | putative ryanodine receptor 44F isoform X4 | 4 | 4 | 9 | 3 | 21.18 |
| ROT73965.1 | hypothetical protein C7M84_007564 | 4 | 4 | 8 | 7 | 17.72 |
| ROT72893.1 | putative transformation/transcription domain-associated protein | 4 | 4 | 7 | 2 | 18.4 |
| ROT80891.1 | hypothetical protein C7M84_000350 | 4 | 4 | 5 | 2 | 10.47 |
| ROT68842.1 | putative vacuolar protein sorting-associated protein 13C | 4 | 4 | 4 | 3 | 9.62 |
| ROT67928.1 | putative tubulin alpha-3 chain-like | 4 | 4 | 4 | 16 | 11.81 |
| ROT75605.1 | hypothetical protein C7M84_005857 | 3 | 3 | 30 | 11 | 67.08 |
| ROT64876.1 | Hexosaminidase domain-containing protein | 3 | 3 | 26 | 14 | 72.11 |
| ROT67290.1 | putative tyrosine-protein phosphatase corkscrew | 3 | 3 | 21 | 8 | 46.6 |
| ROT68219.1 | carboxylase:pyruvate/acetyl-coa/propionyl-CoA | 3 | 3 | 16 | 4 | 64.81 |
| ROT73809.1 | hypothetical protein C7M84_007719 | 3 | 3 | 10 | 5 | 24.35 |
| ROT69904.1 | hypothetical protein C7M84_011862 | 3 | 3 | 10 | 4 | 23.88 |
| ROT69674.1 | hypothetical protein C7M84_012126 | 3 | 3 | 9 | 5 | 20.24 |
| ROT82791.1 | hypothetical protein C7M84_024035 | 3 | 3 | 8 | 5 | 17.88 |
| ROT72146.1 | hypothetical protein C7M84_009480 | 3 | 3 | 6 | 7 | 13.41 |
| ROT83695.1 | putative dystrophin, isoforms A/C/F/G/H isoform | 3 | 3 | 5 | 3 | 11.99 |
| ROT63313.1 | putative transmembrane channel-like protein 3 | 3 | 3 | 5 | 2 | 11.21 |
| ROT75604.1 | hypothetical protein C7M84_005856 | 3 | 3 | 5 | 12 | 11.53 |
| ROT68150.1 | hypothetical protein C7M84_013731 | 3 | 3 | 5 | 3 | 11.33 |
| ROT69221.1 | cardiac muscle actin | 3 | 1 | 5 | 15 | 13.03 |
| ROT61959.1 | hypothetical protein C7M84_020208 | 3 | 3 | 5 | 3 | 11.97 |
| ROT74285.1 | hypothetical protein C7M84_007218 | 3 | 3 | 4 | 1 | 8.65 |
| ROT84375.1 | putative glycine-rich cell wall structural protein 1 | 3 | 3 | 4 | 3 | 9.54 |
| ROT72888.1 | putative nuclear speckle splicing regulatory protein 1-like | 3 | 3 | 4 | 10 | 10.52 |
| ROT60600.1 | putative ATP-dependent RNA helicase DDX24 | 3 | 3 | 4 | 8 | 9.88 |
| ROT68828.1 | putative E3 ubiquitin-protein ligase HERC1 | 3 | 3 | 4 | 2 | 9.99 |
| ROT77602.1 | putative adenomatous polyposis coli protein-like | 3 | 3 | 4 | 2 | 9.12 |
| ROT65495.1 | hypothetical protein C7M84_016526 | 3 | 3 | 4 | 3 | 9.27 |
| ROT78425.1 | centrosomin-like protein | 3 | 3 | 4 | 2 | 9.07 |
| ROT62087.1 | putative RING finger protein nhl-1 | 3 | 3 | 4 | 5 | 8.67 |
| ROT78258.1 | putative vacuolar protein sorting-associated protein 52 | 3 | 3 | 3 | 6 | 6.45 |
| ROT67593.1 | putative coiled-coil domain-containing protein 27 | 3 | 3 | 3 | 2 | 6.35 |
| ROT79227.1 | Low-density lipoprotein receptor-related protein 2 | 3 | 3 | 3 | 3 | 8.39 |
| ROT69824.1 | putative DNA (cytosine-5)-methyltransferase PliMCI-like | 3 | 3 | 3 | 6 | 8.08 |
| ROT63347.1 | hypothetical protein C7M84_018752 | 3 | 3 | 3 | 10 | 6.68 |
| ROT77599.1 | hypothetical protein C7M84_003740 | 3 | 3 | 3 | 3 | 7.3 |
| ROT79757.1 | RING finger protein 31 | 2 | 2 | 55 | 4 | 124.03 |
| ROT64707.1 | Sly1-like protein | 2 | 2 | 47 | 6 | 104.07 |
| ROT68626.1 | Na+/K+-ATPase alpha subunit | 2 | 2 | 45 | 3 | 102.81 |
| ROT63325.1 | hypothetical protein C7M84_018802 | 2 | 2 | 33 | 4 | 72.29 |
| ROT73582.1 | putative janus kinase and microtubule-interacting protein 3 isoform X7 | 2 | 2 | 31 | 3 | 72.14 |
| ROT85541.1 | Ribosome-binding protein 1 | 2 | 2 | 29 | 20 | 66.06 |
| ROT64238.1 | Mitochondrial ribonuclease P protein 1-like | 2 | 2 | 28 | 10 | 76.23 |
| ROT66991.1 | hypothetical protein C7M84_014942 | 2 | 2 | 27 | 5 | 60.73 |
| ROT63507.1 | hypothetical protein C7M84_018607 | 2 | 2 | 23 | 7 | 57.43 |
| ROT61222.1 | putative synaptic vesicular amine transporter | 2 | 2 | 22 | 1 | 48.06 |
| ROT62328.1 | heat shock protein 21 | 2 | 2 | 22 | 3 | 48.09 |
| ROT83109.1 | eukaryotic release factor 1 | 2 | 2 | 18 | 3 | 46.06 |
| ROT63129.1 | hypothetical protein C7M84_018998 | 2 | 2 | 17 | 2 | 38.27 |
| ROT80965.1 | Abhydrolase domain-containing protein 8 | 2 | 2 | 14 | 5 | 37.12 |
| ROT63758.1 | Protein virilizer-like protein | 2 | 2 | 13 | 4 | 35.49 |
| ROT73083.1 | Ral GTPase-activating protein subunit alpha-2 | 2 | 2 | 13 | 3 | 34.14 |
| ROT61312.1 | hypothetical protein C7M84_020917 | 2 | 1 | 12 | 14 | 25.92 |
| ROT62129.1 | hypothetical protein C7M84_020041 | 2 | 2 | 12 | 2 | 29.27 |
| ROT82174.1 | hypothetical protein C7M84_024661 | 2 | 2 | 11 | 14 | 32.74 |
| ROT63707.1 | Alpha-(1,6)-fucosyltransferase | 2 | 2 | 10 | 14 | 28.4 |
| ROT69951.1 | putative FH1/FH2 domain-containing protein 3 | 2 | 2 | 9 | 2 | 20.34 |
| ROT80480.1 | hypothetical protein C7M84_000788 | 2 | 2 | 9 | 4 | 19.5 |
| ROT81450.1 | putative kinesin-like protein KIF12 | 2 | 2 | 8 | 8 | 16.61 |
| ROT66306.1 | Inositol-trisphosphate 3-kinase A | 2 | 2 | 8 | 4 | 18.51 |
| ROT74196.1 | hypothetical protein C7M84_007313 | 2 | 2 | 8 | 1 | 17.45 |
| ROT76400.1 | hypothetical protein C7M84_005013 | 2 | 2 | 8 | 18 | 17.26 |
| ROT82106.1 | Spectrin beta chain | 2 | 2 | 8 | 2 | 18.77 |
| ROT83440.1 | putative calpain-A-like isoform | 2 | 2 | 8 | 12 | 19.91 |
| ROT72257.1 | CUB-serine protease | 2 | 2 | 8 | 5 | 19.87 |
| ROT68666.1 | putative WD repeat-containing protein 59 | 2 | 2 | 7 | 3 | 20.33 |
| ROT67843.1 | putative neurotrimin-like isoform X3 | 2 | 2 | 7 | 19 | 15.48 |
| ROT74734.1 | hypothetical protein C7M84_006759 | 2 | 2 | 7 | 3 | 16.24 |
| ROT82680.1 | WD repeat-containing protein 48 | 2 | 2 | 6 | 8 | 15.49 |
| ROT81932.1 | hypothetical protein C7M84_024919 | 2 | 2 | 6 | 7 | 12.78 |
| ROT60986.1 | hypothetical protein C7M84_ | 2 | 2 | 6 | 3 | 14.41 |
| ROT70122.1 | calcium-translocating P-type ATPase, PMCA-type | 2 | 2 | 6 | 3 | 12.92 |
| ROT79778.1 | Segment polarity protein dishevelled-like protein | 2 | 2 | 6 | 3 | 14.2 |
| ROT83153.1 | hypothetical protein C7M84_023669 | 2 | 2 | 6 | 2 | 13.44 |
| ROT77931.1 | hypothetical protein C7M84_003372 | 2 | 2 | 6 | 3 | 13.35 |
| ROT83500.1 | putative SH3 domain-containing kinase-binding protein 1 | 2 | 2 | 6 | 4 | 13.14 |
| ROT73966.1 | Zinc transporter SLC39A7 | 2 | 2 | 5 | 6 | 12.09 |
| ROT70093.1 | putative glutamate-gated chloride channel-like | 2 | 2 | 5 | 4 | 12.36 |
| ROT63922.1 | hypothetical protein C7M84_018167 | 2 | 2 | 5 | 4 | 11.05 |
| ROT82264.1 | hypothetical protein C7M84_024556 | 2 | 2 | 5 | 4 | 11.15 |
| ROT67836.1 | putative transmembrane protease serine 9-like | 2 | 2 | 5 | 11 | 11.64 |
| ROT79375.1 | putative ATP-binding cassette sub-family A | 2 | 2 | 5 | 5 | 14.35 |
| ROT82054.1 | hypothetical protein C7M84_024781 | 2 | 2 | 5 | 18 | 11.52 |
| ROT77853.1 | Protein disulfide-isomerase A5 | 2 | 2 | 5 | 11 | 14.07 |
| ROT63717.1 | hypothetical protein C7M84_018382 | 2 | 2 | 5 | 3 | 12.26 |
| ROT63411.1 | hypothetical protein C7M84_018721 | 2 | 2 | 5 | 3 | 11.15 |
| ROT81810.1 | putative ubiquitin carboxyl-terminal hydrolase 36 | 2 | 2 | 5 | 2 | 10.9 |
| ROT76353.1 | hypothetical protein C7M84_005051 | 2 | 2 | 5 | 2 | 13.41 |
| ROT77087.1 | putative aminopeptidase N | 2 | 2 | 5 | 5 | 14.39 |
| ROT62920.1 | Tight junction protein ZO-1 | 2 | 2 | 5 | 3 | 11.02 |
| ROT68601.1 | hypothetical protein C7M84_013254 | 2 | 2 | 4 | 5 | 9.13 |
| ROT68917.1 | putative protein transport protein Sec24C | 2 | 2 | 4 | 4 | 9.39 |
| ROT71671.1 | HEAT repeat-containing protein 1 | 2 | 2 | 4 | 1 | 8.94 |
| ROT62266.1 | hypothetical protein C7M84_019906 | 2 | 2 | 4 | 5 | 9.11 |
| ROT74150.1 | hypothetical protein C7M84_007364 | 2 | 2 | 4 | 6 | 9.82 |
| ROT79159.1 | Gamma-interferon-inducible lysosomal thiol reductase | 2 | 2 | 4 | 10 | 9.34 |
| ROT74468.1 | hypothetical protein C7M84_007038 | 2 | 2 | 4 | 7 | 9.7 |
| ROT63217.1 | putative protein furry isoform X1 | 2 | 2 | 4 | 6 | 11.19 |
| ROT69260.1 | hypothetical protein C7M84_012576 | 2 | 2 | 4 | 7 | 6.93 |
| ROT66177.1 | hypothetical protein C7M84_015829 | 2 | 2 | 4 | 4 | 8.78 |
| ROT62064.1 | hypothetical protein C7M84_020102 | 2 | 2 | 4 | 5 | 8.36 |
| ROT71393.1 | putative RNA polymerase II-associated factor 1 | 2 | 2 | 4 | 5 | 8.84 |
| ROT78859.1 | hypothetical protein C7M84_002421 | 2 | 2 | 4 | 2 | 9.31 |
| ROT69861.1 | hypothetical protein C7M84_011907 | 2 | 2 | 4 | 4 | 9.73 |
| ROT73090.1 | hypothetical protein C7M84_008487 | 2 | 2 | 4 | 2 | 8.46 |
| ROT82699.1 | hypothetical protein C7M84_024133 | 2 | 2 | 4 | 6 | 10.08 |
| ROT74447.1 | hypothetical protein C7M84_007050 | 2 | 2 | 4 | 5 | 9.34 |
| ROT72927.1 | putative integrator complex subunit 6-B isoform | 2 | 2 | 4 | 5 | 10.57 |
| ROT84578.1 | hypothetical protein C7M84_022236 | 2 | 2 | 4 | 3 | 9.91 |
| ROT82296.1 | hypothetical protein C7M84_024534 | 2 | 2 | 4 | 1 | 8.5 |
| ROT83496.1 | Phosphoinositide 3-kinase regulatory subunit 4 | 2 | 2 | 4 | 4 | 11.21 |
| ROT83218.1 | Cell surface A33 antigen | 2 | 2 | 4 | 3 | 8.67 |
| ROT74830.1 | hypothetical protein C7M84_006659 | 2 | 2 | 4 | 5 | 9.6 |
| ROT70035.1 | hypothetical protein C7M84_011713 | 2 | 2 | 4 | 4 | 8.58 |
| ROT69632.1 | putative receptor-type tyrosine-protein phosphatase S | 2 | 2 | 3 | 3 | 7.8 |
| ROT80053.1 | DNA replication licensing factor MCM4 | 2 | 2 | 3 | 5 | 7.68 |
| ROT68028.1 | putative deoxyribonuclease-1 | 2 | 2 | 3 | 13 | 8.91 |
| ROT75691.1 | putative alkylglycerol monooxygenase-like | 2 | 2 | 3 | 11 | 6.05 |
| ROT66208.1 | Neurobeachin | 2 | 2 | 3 | 1 | 7.59 |
| ROT62482.1 | putative zinc finger BED domain-containing protein | 2 | 2 | 3 | 6 | 6.21 |
| ROT64003.1 | embryonic gonad protein | 2 | 2 | 3 | 3 | 6.34 |
| ROT62495.1 | Facilitated trehalose transporter Tret1 | 2 | 2 | 3 | 4 | 7.5 |
| ROT65240.1 | Translational activator GCN1 | 2 | 2 | 3 | 2 | 8.35 |
| ROT75338.1 | putative ATP-dependent RNA helicase Pl10 | 2 | 2 | 3 | 4 | 6.81 |
| ROT65108.1 | hypothetical protein C7M84_016943 | 2 | 2 | 3 | 2 | 6.93 |
| ROT73858.1 | putative CCR4-NOT transcription complex subunit 4 isoform X3 | 2 | 2 | 3 | 7 | 7.4 |
| ROT84288.1 | hypothetical protein C7M84_ | 2 | 2 | 3 | 15 | 7.69 |
| ROT71794.1 | putative fork head domain-containing protein FD5 | 2 | 2 | 3 | 12 | 8.22 |
| ROT63672.1 | putative uncharacterized protein K02A2.6-like | 2 | 2 | 3 | 3 | 6.05 |
| ROT79351.1 | kinesin heavy chain | 2 | 2 | 3 | 5 | 7.68 |
| ROT70839.1 | Carbohydrate sulfotransferase 11 | 2 | 2 | 3 | 7 | 7.24 |
| ROT65480.1 | target of rapamycin | 2 | 2 | 3 | 2 | 7.94 |
| ROT75979.1 | Cj-cadherin | 2 | 2 | 3 | 2 | 7.46 |
| ROT72921.1 | hypothetical protein C7M84_008668 | 2 | 2 | 3 | 3 | 6.31 |
| ROT64525.1 | hypothetical protein C7M84_017531 | 2 | 2 | 3 | 2 | 7.28 |
| ROT70240.1 | Filamin-A | 2 | 2 | 3 | 6 | 6.96 |
| ROT75997.1 | hypothetical protein C7M84_005431 | 2 | 2 | 3 | 3 | 6.41 |
| ROT80795.1 | putative serine/threonine-protein phosphatase 6 regulatory ankyrin repeat subunit B-like | 2 | 2 | 3 | 2 | 8.2 |
| ROT75068.1 | hypothetical protein C7M84_006409 | 2 | 2 | 3 | 1 | 6.81 |
| ROT66270.1 | hypothetical protein C7M84_015732 | 2 | 2 | 3 | 1 | 6.37 |
| ROT66242.1 | putative rootletin | 2 | 2 | 3 | 1 | 6.22 |
| ROT82303.1 | Islet cell autoantigen 1 | 2 | 2 | 3 | 8 | 7.22 |
| ROT75125.1 | putative myosin light chain kinase | 2 | 2 | 3 | 1 | 6.42 |
| ROT81616.1 | hypothetical protein C7M84_025230 | 2 | 2 | 3 | 2 | 9.21 |
| ROT70887.1 | putative titin | 2 | 2 | 3 | 1 | 7.68 |
| ROT62117.1 | putative targeting protein for Xklp2-like | 2 | 2 | 3 | 4 | 6.32 |
| ROT78667.1 | putative zinc finger protein 85-like | 2 | 2 | 3 | 12 | 7.78 |
| ROT76182.1 | putative dentin sialophosphoprotein | 2 | 2 | 3 | 5 | 7.86 |
| ROT63594.1 | heat shock protein 21 | 2 | 2 | 3 | 6 | 6.43 |
| ROT81988.1 | hypothetical protein C7M84_024842 | 2 | 2 | 3 | 7 | 7.57 |
| ROT76009.1 | putative vascular endothelial growth factor receptor 1 | 2 | 2 | 3 | 4 | 9.14 |
| ROT65254.1 | hypothetical protein C7M84_016794 | 2 | 2 | 3 | 2 | 6.85 |
| ROT77979.1 | kinetoplast-associated protein-like protein | 2 | 2 | 3 | 2 | 6.12 |
| ROT77895.1 | hypothetical protein C7M84_003410 | 2 | 2 | 3 | 8 | 6.8 |
| ROT63526.1 | hypothetical protein C7M84_018595 | 2 | 2 | 3 | 5 | 7.32 |
| ROT84248.1 | hypothetical protein C7M84_022565 | 2 | 2 | 3 | 12 | 8.66 |
| ROT80200.1 | Methyltransferase-like 25 | 2 | 2 | 3 | 7 | 7.26 |
| ROT68296.1 | hypothetical protein C7M84_013571 | 2 | 2 | 3 | 1 | 6.49 |
| ROT77325.1 | putative DNA topoisomerase 2 isoform X2 | 2 | 2 | 3 | 2 | 6.84 |
| ROT65124.1 | neurofilament protein | 2 | 2 | 3 | 2 | 7.9 |
| ROT60844.1 | alpha-2-macroglobulin | 2 | 2 | 3 | 5 | 7.12 |
| ROT65248.1 | hypothetical protein C7M84_016803 | 2 | 2 | 2 | 2 | 5.79 |
| ROT73366.1 | hypothetical protein C7M84_008203 | 2 | 2 | 2 | 8 | 6.27 |
| ROT67891.1 | hypothetical protein C7M84_014008 | 2 | 2 | 2 | 1 | 4.3 |
| ROT62352.1 | Guanine nucleotide exchange factor DBS | 2 | 2 | 2 | 8 | 4.69 |
| ROT65923.1 | hypothetical protein C7M84_016079 | 2 | 2 | 2 | 1 | 4.17 |
| ROT74205.1 | hypothetical protein C7M84_007300 | 2 | 2 | 2 | 12 | 4.26 |
| ROT68603.1 | putative collagen alpha-2(IV) chain-like | 2 | 2 | 2 | 14 | 4.99 |
| ROT63039.1 | hypothetical protein C7M84_019087 | 2 | 2 | 2 | 2 | 4.32 |
| ROT84958.1 | putative piggyBac transposable element-derived protein 4 | 2 | 2 | 2 | 9 | 4.95 |
| ROT70371.1 | hypothetical protein C7M84_011352 | 2 | 2 | 2 | 1 | 4.1 |
| ROT70986.1 | hypothetical protein C7M84_010710 | 2 | 2 | 2 | 6 | 5.7 |
| ROT66243.1 | hypothetical protein C7M84_015765 | 2 | 2 | 2 | 7 | 5.78 |
| ROT70835.1 | putative A-kinase anchor protein 9 | 2 | 2 | 2 | 1 | 4.34 |
| ROT78978.1 | hypothetical protein C7M84_002303 | 2 | 2 | 2 | 7 | 4.66 |
| ROT85633.1 | hypothetical protein C7M84_009791 | 2 | 2 | 2 | 2 | 4.15 |
| ROT76862.1 | putative tyrosine-protein kinase-like otk | 2 | 2 | 2 | 2 | 4.26 |
| ROT63098.1 | hypothetical protein C7M84_019029 | 2 | 2 | 2 | 2 | 5.01 |
| ROT65209.1 | Replicase polyprotein 1a | 2 | 2 | 2 | 8 | 5.18 |
| ROT63016.1 | hypothetical protein C7M84_019095 | 2 | 2 | 2 | 1 | 4.18 |
| ROT60610.1 | hypothetical protein C7M84_021861 | 2 | 2 | 2 | 3 | 4.23 |
| ROT62066.1 | hypothetical protein C7M84_020104 | 2 | 2 | 2 | 9 | 5.09 |
| ROT76260.1 | putative leucine-rich repeat-containing protein 45-like | 2 | 2 | 2 | 5 | 4.91 |
| ROT63015.1 | hypothetical protein C7M84_019120 | 2 | 2 | 2 | 8 | 5.74 |
| ROT69788.1 | Flotillin-1 | 2 | 2 | 2 | 9 | 5.36 |
| ROT63339.1 | hypothetical protein C7M84_018790 | 2 | 2 | 2 | 1 | 5.43 |
| ROT83614.1 | putative WD repeat-containing protein 19-like | 2 | 2 | 2 | 2 | 5.16 |
| ROT61251.1 | hypothetical protein C7M84_021011 | 2 | 2 | 2 | 6 | 4.55 |
| ROT65912.1 | hypothetical protein C7M84_016110 | 2 | 2 | 2 | 6 | 4.29 |
| ROT63188.1 | hypothetical protein C7M84_018942 | 2 | 2 | 2 | 1 | 4.52 |
| ROT65473.1 | hypothetical protein C7M84_016552 | 2 | 2 | 2 | 1 | 4.22 |
| ROT62586.1 | hypothetical protein C7M84_019571 | 2 | 2 | 2 | 2 | 4.27 |
| ROT68844.1 | heat shock protein 70 | 2 | 2 | 2 | 5 | 5.55 |
| ROT81869.1 | putative 5-hydroxytryptamine receptor 1A-alpha-like | 2 | 2 | 2 | 11 | 5.72 |
| ROT62095.1 | hypothetical protein C7M84_020076 | 2 | 2 | 2 | 4 | 4.13 |
| ROT76935.1 | putative kinesin-like protein KIF14 | 2 | 2 | 2 | 3 | 5.09 |
| ROT77276.1 | Zinc finger FYVE domain-containing protein 26 | 2 | 2 | 2 | 2 | 5.55 |
| ROT68116.1 | hypothetical protein C7M84_013789 | 2 | 2 | 2 | 3 | 5.11 |
| ROT67723.1 | putative cuticle protein | 2 | 2 | 2 | 12 | 5.39 |
| ROT71829.1 | hypothetical protein C7M84_009835 | 2 | 2 | 2 | 3 | 4.22 |
| ROT65760.1 | hypothetical protein C7M84_016259 | 2 | 2 | 2 | 5 | 4.7 |
| ROT65070.1 | hypothetical protein C7M84_016982 | 2 | 2 | 2 | 7 | 5.09 |
| ROT80561.1 | Vitamin D3 receptor B | 2 | 2 | 2 | 4 | 4.97 |
| ROT72463.1 | putative transcription elongation factor SPT6-like | 2 | 2 | 2 | 1 | 4.21 |
| ROT68800.1 | hypothetical protein C7M84_013048 | 2 | 2 | 2 | 2 | 4.1 |
| ROT77539.1 | Cell division cycle protein 27-like protein, partial | 2 | 2 | 2 | 5 | 5.41 |
| ROT67659.1 | putative EH domain-binding protein 1-like | 2 | 2 | 2 | 8 | 5.76 |
| ROT85261.1 | putative activating transcription factor 7-interacting protein 1 | 2 | 2 | 2 | 6 | 4.98 |
| ROT74448.1 | putative ankyrin-1-like | 2 | 2 | 2 | 6 | 4.27 |
| ROT63731.1 | nesprin-1-like | 2 | 2 | 2 | 1 | 4.74 |
| ROT66486.1 | Leucine-rich PPR motif-containing protein | 2 | 2 | 2 | 2 | 4.18 |
| ROT74350.1 | hypothetical protein C7M84_007162 | 2 | 2 | 2 | 2 | 4.04 |
| ROT76274.1 | hypothetical protein C7M84_005168 | 2 | 2 | 2 | 1 | 4.19 |
| ROT82210.1 | hypothetical protein C7M84_024633 | 2 | 2 | 2 | 5 | 5.51 |
| ROT80144.1 | hypothetical protein C7M84_001143 | 2 | 2 | 2 | 0 | 4.13 |
| ROT81449.1 | Dynein heavy chain, cytoplasmic | 2 | 2 | 2 | 1 | 4.26 |
| ROT64520.1 | hypothetical protein C7M84_017548 | 2 | 2 | 2 | 5 | 5.41 |
| ROT61433.1 | hypothetical protein C7M84_020785 | 2 | 2 | 2 | 7 | 5.38 |
| ROT85098.1 | putative DNA mismatch repair protein Mlh3 | 2 | 2 | 2 | 7 | 4.84 |
| ROT65380.1 | hypothetical protein C7M84_016660 | 2 | 2 | 2 | 1 | 5.12 |
| ROT66061.1 | hypothetical protein C7M84_015957 | 2 | 2 | 2 | 2 | 4.25 |
| ROT74813.1 | cytochrome P450 CYP6BK17 | 2 | 2 | 2 | 7 | 4.23 |
| ROT82847.1 | FERM and PDZ domain-containing protein 4 | 2 | 2 | 2 | 1 | 4.07 |
| ROT68712.1 | neural-cadherin-like | 2 | 2 | 2 | 8 | 4.74 |
| ROT79530.1 | 26S proteasome non-ATPase regulatory subunit 2 | 2 | 2 | 2 | 4 | 4.11 |
| ROT68521.1 | heat shock protein 60 | 2 | 2 | 2 | 8 | 5.81 |
| ROT83492.1 | hypothetical protein C7M84_023335 | 2 | 2 | 2 | 5 | 5.4 |
| ROT81135.1 | putative DNA topoisomerase 3-beta-1 | 2 | 2 | 2 | 3 | 4.04 |
| ROT80212.1 | hypothetical protein C7M84_001063 | 2 | 2 | 2 | 8 | 5.27 |
| ROT66000.1 | hypothetical protein C7M84_016007 | 2 | 2 | 2 | 1 | 5.68 |
| ROT61574.1 | hypothetical protein C7M84_020620 | 2 | 2 | 2 | 10 | 6.33 |
| ROT75811.1 | Protein ELYS | 2 | 2 | 2 | 1 | 5.11 |
| ROT61796.1 | hypothetical protein C7M84_020393 | 2 | 2 | 2 | 8 | 5.13 |
| ROT62775.1 | hypothetical protein C7M84_019358 | 2 | 2 | 2 | 7 | 4.94 |
| ROT83639.1 | hypothetical protein C7M84_023172 | 2 | 2 | 2 | 3 | 4.14 |
| ROT67017.1 | hypothetical protein C7M84_014926 | 2 | 2 | 2 | 2 | 4.22 |
| ROT81577.1 | hypothetical protein C7M84_025250 | 2 | 2 | 2 | 3 | 4.19 |
| ROT65949.1 | hypothetical protein C7M84_016076 | 2 | 2 | 2 | 1 | 4.55 |
| ROT65381.1 | hypothetical protein C7M84_016661 | 2 | 2 | 2 | 1 | 4.25 |
| ROT65749.1 | Cj-cadherin | 2 | 2 | 2 | 5 | 5.98 |
| ROT68581.1 | Rad50 | 2 | 2 | 2 | 3 | 4.1 |
| ROT63491.1 | hypothetical protein C7M84_018614 | 2 | 2 | 2 | 4 | 5.8 |
| ROT85129.1 | myosin Va | 2 | 2 | 2 | 2 | 4.35 |
| ROT78252.1 | hypothetical protein C7M84_003038 | 2 | 2 | 2 | 5 | 5.39 |
| ROT73463.1 | hypothetical protein C7M84_008128 | 2 | 2 | 2 | 7 | 4.05 |
| ROT69633.1 | hypothetical protein C7M84_012137 | 2 | 2 | 2 | 2 | 4.24 |
| ROT71003.1 | hypothetical protein C7M84_010704 | 2 | 2 | 2 | 2 | 6.13 |
| ROT65869.1 | hypothetical protein C7M84_016147 | 2 | 2 | 2 | 3 | 5.99 |
| ROT76701.1 | putative ATP-binding cassette sub-family G member 4 | 2 | 2 | 2 | 5 | 5.28 |
| ROT71962.1 | hypothetical protein C7M84_009672 | 2 | 2 | 2 | 5 | 4.89 |
| ROT74041.1 | putative echinoderm microtubule-associated protein | 2 | 2 | 2 | 3 | 5.32 |

**Table S2 The binding sites between *Pv*ATP1A and VP28 as determined by molecular docking**

| Chain 1 | Residue | Chain 2 | Residue | Interaction type |
| --- | --- | --- | --- | --- |
| *Pv*ATP1A | Glu137.OE2 | VP28 | Asn154.N | Hydrogen bond interaction |
| *Pv*ATP1A | His897.ND1 | VP28 | Asp49.OD2 | Salt bridge |
| *Pv*ATP1A | Glu903.OE1 | VP28 | Gln138.NE2 | Hydrogen bond interaction |
| *Pv*ATP1A | Arg904.NH2 | VP28 | Gly136.O | Hydrogen bond interaction |
| *Pv*ATP1A | Arg904.NH2 | VP28 | Tyr193.O | Hydrogen bond interaction |
| *Pv*ATP1A | Glu914.OE1 | VP28 | Ser199.OG | Hydrogen bond interaction |
| *Pv*ATP1A | Glu914.OE2 | VP28 | Thr201.OG1 | Hydrogen bond interaction |
| *Pv*ATP1A | His916.CE1 | VP28 | Thr46.OG1 | Hydrogen bond interaction |
| *Pv*ATP1A | His916.NE2 | VP28 | Asn47.OD1 | Hydrogen bond interaction |

**Table S3 The primer sequences used in this study**

| **Name** | **Sequence (5'-3')** |
| --- | --- |
| GST-BirA*-F | TCCAGGGGCCCCTGGGATCCATGTTCAAGAACCTGATCTGGC |
| GST-BirA*-R | CCCGGGAATTCCGGGGATCCTTAGCTTCTTCTCAGGCTGAAC |
| GST-BirA*-VP28-F | GCCTGAGAAGAAGCCTCGAGATGGATCTTTCTTTCACTCTTT |
| GST-BirA*-VP28-R | TGGTGGTGGTGGTGCTCGAGTTACTCGGTCTCAGTGCCAGAG |
| *Pv*ATP1A-F | ATGGCCGATACGGGCAGAAC |
| *Pv*ATP1A-R | TTAATAGTAGGTCTCCAGTT |
| dsEGFP-T7F | GGATCCTAATACGACTCACTATAGGCGTAAACGGCCACAAGTT |
| dsEGFP-T7R | GGATCCTAATACGACTCACTATAGGTTCACCTTGATGCCGTTC |
| ds*Pv*Glut1-T7F | GGATCCTAATACGACTCACTATAGGAGGTTTGGCAGAAAGAAGGG |
| ds*Pv*Glut1-T7R | GGATCCTAATACGACTCACTATAGGGCTTCATCCTCTAACTGGCGTG |
| ds*Pv*pIgR-T7F | GGATCCTAATACGACTCACTATAGGGCGTAAATGAACCAAACAACAG |
| ds*Pv*pIgR-T7R | GGATCCTAATACGACTCACTATAGGTAAGAACCAGCGAATACCCACA |
| ds*Pv*ATP1A-T7F | GGATCCTAATACGACTCACTATAGGAAGCATCGCCGTTTACTTC |
| ds*Pv*ATP1A-T7R | GGATCCTAATACGACTCACTATAGGCAATGTGCCTGAGGGTCTG |
| ds*Pv*β-intergin-T7F | GGATCCTAATACGACTCACTATAGGGCCGCTGAGTGATGTTTCG |
| ds*Pv*β-intergin-T7R | GGATCCTAATACGACTCACTATAGGGGCGCTGAGGACTTTTTCC |
| dsEGFP-F | CGTAAACGGCCACAAGTT |
| dsEGFP-R | TTCACCTTGATGCCGTTC |
| ds*Pv*Glut1-F | AGGTTTGGCAGAAAGAAGGG |
| ds*Pv*Glut1-R | GCTTCATCCTCTAACTGGCGTG |
| ds*Pv*pIgR-F | GCGTAAATGAACCAAACAACAG |
| ds*Pv*pIgR-R | TAAGAACCAGCGAATACCCACA |
| ds*Pv*ATP1A-F | AAGCATCGCCGTTTACTTC |
| ds*Pv*ATP1A-R | CAATGTGCCTGAGGGTCTG |
| ds*Pv*β-integrin-F | GCCGCTGAGTGATGTTTCG |
| ds*Pv*β-integrin-R | GGCGCTGAGGACTTTTTCC |
| qVP28-F | AAACCTCCGCATTCCTGTGA |
| qVP28-R | TCCGCATCTTCTTCCTTCAT |
| qIE1-F | GCACAACAACAGACCCTACCC |
| qIE1-R | GAAATACGACATAGCACCTCCAC |
| q*Pv*EF-1α-F | TATGCTCCTTTTGGACGTTTTGC |
| q*Pv*EF-1α-R | CCTTTTCTGCGGCCTTGGTAG |
| q*Pv*Glut1-F | GCTTATTCGGGTTTAACGTGC |
| q*Pv*Glut1-R | AAATCCTGCTTGCTGCCTTC |
| q*Pv*pIgR-F | GCACTTACTACCAGATTCGTCCAG |
| q*Pv*pIgR-R | CAGCAATCCTCAAGGTAGCAGT |
| q*Pv*ATP1A-F | AGCAAGGCCATCAACGATCT |
| q*Pv*ATP1A-R | GCCCACTGCACAATCACAAT |
| q*Pv*β-integrin-F | TACGACTACCCTTCTGTGGCGC |
| q*Pv*β-integrin-R | GTGTCTGTCAGGTTGTCGCTCA |

| **Name** | **Sequence (N to C)^a^** |
| --- | --- |
| pEGFP | MVSKGEELFT |
| pATP1A-ER1 | ETAAEEEPNKD |
| pATP1A-ER2 | HWLDA |
| pATP1A-ER3 | ASVPLPLG |
| pATP1A-ER4 | MAENGFLPPHLFGLRERWDSKAINDLEDHYGQEWTFHDRK |
| pATP1A-ER5 | MDKGLRMYPLK |

**Table S4 The synthetic peptide sequences used in this study**

^a^ These peptides were labeled with biotin at the N-terminus
